# Disruption of YY1-EZH2 Interaction Using Synthetic Peptides Inhibits Breast Cancer Development

**DOI:** 10.3390/cancers13102402

**Published:** 2021-05-16

**Authors:** Cheng Yi, Guangyue Li, Wenmeng Wang, Yixuan Sun, Yueling Zhang, Chen Zhong, Daniel B. Stovall, Dangdang Li, Jinming Shi, Guangchao Sui

**Affiliations:** 1 Key Laboratory of Saline-Alkali Vegetation Ecology Restoration, Ministry of Education, College of Life Science, Northeast Forestry University, Harbin 150040, China; yc6659689@nefu.edu.cn (C.Y.); guangyuel@nefu.edu.cn (G.L.); wangwenmeng@nefu.edu.cn (W.W.); yixuansun@nefu.edu.cn (Y.S.); lidd@nefu.edu.cn (D.L.); 2 State Key Laboratory of Veterinary Biotechnology, Harbin Veterinary Research Institute, Chinese Academy of Agricultural Sciences, Harbin 150069, China; zhangyueling@caas.cn; 3 State Key Laboratory of Genetic Engineering, School of Life Sciences, Fudan University, Shanghai 200438, China; zhongchen@fudan.edu.cn; 4 College of Arts and Sciences, Winthrop University, Rock Hill, SC 29733, USA; stovalld@winthrop.edu

**Keywords:** EZH2, YY1, YPB, OPB, breast cancer, PTENP1

## Abstract

**Simple Summary:**

Both Yin Yang 1 (YY1) and enhancer of zeste homolog 2 (EZH2) are oncogenes with overexpressed statuses in cancers. As a transcription factor, YY1 recruits EZH2 through its oncoprotein binding (OPB) domain to repress gene expression. In this study, we identified the interaction domain of YY1 on EZH2 protein with amino acids 493–519, named the YY1 protein binding (YPB) domain. Synthetic peptides using YPB and OPB domain sequences effectively blocked endogenous YY1-EZH2 interaction. Functionally, YPB and OPB peptides could efficiently inhibit the proliferation of breast cancer cells, promote their apoptosis, and reduce tumor growth in a xenograft mouse model. Using chromatin immunoprecipitation DNA sequencing (ChIP-seq) analysis, we discovered that YPB and OPB peptides could interfere with H3K27 trimethylation of multiple genes. Eventually, we identified that YPB and OPB peptides primarily targeted the PTENP1 gene and validated its importance in the anticancer activity of the two peptides.

**Abstract:**

Enhancer of zeste homolog 2 (EZH2) is a methyltransferase to mediate lysine 27 trimethylation in histone H3 (i.e., H3K27me3) and repress gene expression. In solid tumors, EZH2 promotes oncogenesis and is considered a therapeutic target. As a transcription factor, Yin Yang 1 (YY1) recruits EZH2 through its oncoprotein binding (OPB) domain to establish gene repression. In this study, we mapped the YY1 protein binding (YPB) domain on EZH2 to a region of 27 amino acids. Both YPB and OPB domain synthetic peptides could disrupt YY1EZH2 interaction, markedly reduce breast cancer cell viability, and efficiently inhibit tumor growth in a xenograft mouse model. We analyzed MDA-MB-231 cells treated with YPB, OPB, and control peptides by chromatin immunoprecipitation DNA sequencing (ChIP-seq) using an antibody against H3K27me3. YPB and OPB treatments altered H3K27me3 on 465 and 1137 genes, respectively, compared to the control. Of these genes, 145 overlapped between the two peptides. Among them, PTENP1, the PTEN pseudogene, showed reduced H3K27me3 signal when treated by either YPB or OPB peptide. Consistently, the two peptides enhanced both PTENP1 and PTEN expression with concomitantly reduced AKT activation. Further studies validated PTENP1′s contribution to the anticancer activity of YPB and OPB peptides.

## 1. Introduction

Epigenetic regulation plays a key role in cell proliferation, differentiation, and embryonic development. Meanwhile, deregulated epigenetic processes cause and maintain the aberrant gene expression profiles of cancer cells, and contribute to cancer development and progression [1]. Unlike genetic mutations, epigenetic alterations are generally reversible, and thus adjusting deregulated epigenetics can potentially restore normal gene expression and attenuate cancer progression. Various therapeutics modulating epigenetic processes, such as inhibitors of DNA methyltransferases and histone deacetylases, have been developed and used in the treatment of various cancers [2].

Histone modifications represent an important regulatory approach in controlling gene expression through the actions of different protein modifiers. Among them, the tri-methylation of lysine 27 on histone H3 (i.e., H3K27me3) is a hallmark of gene repression and specifically mediated by the enhancer of zeste homolog 2 (EZH2), a histone methyltransferase [3]. As an integral and catalytic component of the Polycomb repressive complex 2 (PRC2), EZH2 plays a critical role in cell proliferation, embryonic development, stem cell maintenance and cell lineage specification [4,5]. Increasingly evidence indicates that EZH2 is overexpressed in many solid tumors, including breast and prostate cancers, and correlated with poor clinical outcomes among patients [6]. Consistently, EZH2 has been demonstrated to promote cancer cell proliferation, migration, invasion, and metastasis through regulating the expression of a large array of target genes [7,8,9,10]. Thus, EZH2 is considered as be a proto-oncogene in solid tumors [11]. In breast cancer, EZH2 also showed increasing expression compared to normal cells and tissues, and could promote oncogenic transformation of primary mammary cells [12,13]. Therefore, EZH2 has been recognized as a bona fide target in cancer therapies, and many EZH2 inhibitors have been evaluated in clinical trials, such as GSK126 and EPZ005687 [14,15]. Like many other transcriptional cofactors, EZH2 does not directly bind DNA, but can be recruited by other molecules to its target promoters, where it exerts its regulatory activity. Actually, Yin Yang 1 (YY1) was the first transcription factor identified to recruit PRC2 and mediate EZH2 activity [16]. Other transcription factors or chromatin binding proteins, such as TCF1 and CDYL, could also help EZH2 bind its target genes to regulate their expression [17,18]. Since the discovery that long noncoding RNA HOTAIR could recruit EZH2 and other histone modifiers to chromatin [19], understanding of the regulation of EZH2′s epigenetic activity has been greatly extended. In recent years, the RNA binding affinity of EZH2 and its recruitment to chromatin by many other lncRNAs, including PVT1, MALAT1, HOXD-AS1, FOXC2-AS1, and GATA6-AS1, have been frequently reported [20,21,22,23,24].

YY1 has been demonstrated as a transcription factor to either activate or repress gene expression through recruiting different histone modifiers [25]. At the transcriptional level, YY1 was reported to activate the expression of various genes with oncogenic or proliferative functions, including MYC, HER2, CDC6, and TGFβ [25]. Additionally, YY1 also promotes oncogenesis through regulation independent of its transcriptional activity. We reported that YY1 enhanced MDM2-mediated p53 ubiquitination and degradation [26], which was validated by several other studies [27,28,29]. YY1 could also promote AKT activation through binding to its PH domain and facilitating mTORC2-mediated AKT phosphorylation at S473 [30]. Importantly, the residues (201–226) of YY1 are responsible for the binding of EZH2, MDM2, and AKT [30], and also involved in interacting with the viral oncoprotein E1A [31]; thus, we named this region the oncoprotein binding (OPB) domain [30]. YY1-mediated EZH2 activity is essential to gene expression; thus, we predicted that disruption of their interaction could cause perturbation in breast cancer cells and thus inhibit cancer progression. As we reported that both YY1 and EZH2 were overexpressed in breast cancer cells and cancers versus normal mammary samples, blocking EZH2 recruitment by YY1 would cause more pronounced adverse effects in tumor cells than those in normal cells, implicating the relatively low side effects of this strategy.

As potent anticancer agents, the primary advantage of peptides is their high specificity compared to traditional therapeutics. To design peptides that can specifically disrupt protein-protein binding, we need prerequisite knowledge of protein interactions. In this study, we carefully mapped the YY1-binding domain on EZH2 and identified a region consisting of 27 residues as the YY1 protein binding (YPB) domain. We observed that a synthetic peptide based on the YPB domain, and another peptide using the OPB sequence, could efficiently reduce breast cancer cell viability in vitro and inhibit tumor growth in a xenograft tumor model, compared to a control peptide. We also employed chromatin immunoprecipitation DNA sequencing (ChIP-seq) to inspect the alterations of H3K27me3 in response to peptide treatment and determined that PTENP1 acted as a primary target through which these peptides exert their anticancer activities.

## 2. Materials and Methods

### 2.1. Cell Culture and Transfection

MDA-MB-231 and MDA-MB-453 cells were cultured in RPMI-1640 supplemented with 10% fetal bovine serum (FBS) (Biological Industries USA, Inc., Cromwell, CT, USA). MCF-10A and MCF-7 cells were cultured in DMEM/F12 supplemented with 10% FBS, 1% penicillin/streptomycin (HyClone, Logan, UT, USA), 0.5 mg/mL Hydrocortisone (Sigma, St. Louis, MO, USA), 100 ng/mL cholera toxin (Sigma), 10 mg/mL insulin (Sigma), and 20 ng/mL recombinant human EGF (Sigma). The cells were cultured in a 37 °C incubator supplied with 5% CO_2_. Lipofectamine 2000 (Invitrogen, Carlsbad, CA, USA) was used in transient transfection of the cells following the procedure provided by the manufacturer.

### 2.2. Plasmids

HA-tagged expression vectors of wild-type (wt) and mutant YY1 were constructed by amplifying and subcloning the corresponding fragments of the YY1 coding region into the pcDNA3 vector. To generate EZH2 mutants, different regions of its coding sequence (1–251, 252–384, 385–618, 619–751, 385–465, 465–545, 545–618, 465–492, and 465–519) were amplified and subcloned into pGEX-4T-1 and pSL4-3×Flag plasmids to construct vectors expressing recombinant GST-EZH2 and 3×Flag-EZH2 mutants, respectively. To generate vectors expressing 3×Flag-YPB, -OPB, and -Cont (with a scrambled sequence of WHPQPKKLRCSKSDAAKRRLRGKKIKH) peptides, their coding sequences were individually subcloned into a lentiviral vector pSL4 using the CMV promoter to drive the expression of 3×Flag-YPB-2A1-EGFP, 3×Flag-OPB-2A1-EGFP, and 3×Flag-Cont-2A1-EGFP. The 2A1 sequence in mRNA could cause “ribosomal skipping”, leading to the translation of separate YPB and EGFP products [32]. Two shRNAs against PTENP1 (shPTENP1-1 and shPTENP1-2, with target sequences of GCCAAATCTAATTACAGAGTT and GGGAGGACAAGTTCATGTATT, respectively), and one control shRNA (with a scrambled target sequence of GGGACTACTCTATTACGTCATT) driven by the U6 promoter were generated in a lentiviral vector with a puromycin selection marker as we previously described [33,34]. In addition, we also generated lentiviruses expressing two shRNAs (shYY1-1 and shYY1-2, with target sequences of GGGAGCAGAAGCAGGTGCAGTT and GCTCACCTGTTGCTTACAATT, respectively), and two EZH2 (shEZH2-1 and shEZH2-2, with target sequences of GAATACTGTGGAGAGATTATT and GGTGATCACAGGA-TAGGTATT, respectively).

Lentivirus packaging also followed a previously reported procedure [34]. Cells infected by lentivirus were cultured in medium containing 1.0 µg/µL of puromycin for at least 2 days followed by quantitative analyses.

### 2.3. Antibodies

Antibodies used were as follows: antibodies against caspase 3 (cat# 9665), cleaved caspase 3 (cat# 9664), PARP (cat# 9532), cleaved PARP (cat# 5625), GAPDH (cat# 51332S), H3k27me3 (cat# 9733S), PTEN (cat# 9188S), AKT (cat# 4685S), pAKT-Ser473 (cat# 4060S), pAKT-Thr308 (cat# 13038S), and EZH2 (cat# 5246S) were purchased from Cell Signaling Technology, Inc., Danvers, MA, USA; antibodies against Flag (cat# F3165), GST (cat# PA1-982A), and Ki-67 (cat# 550609) were purchased from Sigma-Aldrich, St. Louis, MO, USA, Thermo Fisher Scientific, Waltham, MA, USA, and BD Pharmingen, San Diego, CA, USA, respectively; antibodies against YY1 (H-10, cat# sc-7341, sc-281, and c-20) were purchased from Santa Cruz Biotechnology, Santa Cruz, CA, USA.

### 2.4. Synthetic Peptide Treatment and Quantitative PCR (qPCR)

All peptides were synthesized by ChinaPeptides Co., Ltd. (Shanghai, China). Total RNA was extracted from cell and tissue samples using the Trizol reagent (Invitrogen). In qPCR, LightCycler 480 SYBR Green I Master from Roche was employed, with random hexamers as primers in reverse transcription if needed. Primers used in qPCR included: human PTENP1, forward: TCAGAACATGGCATACACCAA, and reverse: TGATGACGTCCGATTTTTCA; human PTEN, forward: CTTACAGTTGGGCCCTGTACCATCC, and reverse: TTTGATGCTGCCGGTAAACTCCACT; human YY1, forward: ATGCTAAGGCCAAAAACAACCAGTG, and reverse: TGAAAC-GAGATTACAGAGCAAGATA; human EZH2, forward: GAGACAGCTCAA-GAGGTTCAGAC, and reverse: CACTTATGGGTACTGAAGCAACTG; and human GAPDH: forward: GTCTCCTCTGACTTCAACAGCG, and reverse: ACCACCCTGTTGCTGTAGCCAA.

### 2.5. Surface Plasmon Resonance (SPR)

SPR studies were performed using the Biacore T200 biosensor (GE Healthsciences). About 12,000 resonance units (RU) of His×6-YY1 were immobilized by amine coupling to the carboxymethylated dextran matrix of the CM5 Chip (GE Healthcare). Purified GST-EZH2 mutant proteins, GST, and YPB peptides were serially diluted into concentrations of 62.5, 125, 250, 500, and 1000 nM for injection. The samples flowed over the chip surface, with the response units measured at a single cycle. The binding kinetics were analyzed using 1:1 binding model with Biacore T200 Evaluation software, version 2.0.

### 2.6. Co-Immunoprecipitation (Co-IP) and Western Blot

Immunoprecipitations were performed as described previously [35]. Cells were harvested and lysed in cell lysis buffer (50 mM Tris⋅HCl, pH 7.5, 150 mM NaCl, 1% Nonidet P-40, 0.5% sodium deoxycholate, and 1% protease inhibitor cocktails, Sigma-Aldrich). Cell lysates were centrifuged at 14,000 rpm, 4 °C for 15 min, and the supernatant was incubated with 2 µg of an antibody and 50 µL of Protein A/G beads (Thermo Fisher Scientific) at 4 °C overnight. The beads were washed at least 5 times using the lysis buffer, and the precipitated proteins were used for further analyses. In Western blot studies, protein concentrations of samples were determined using the Bradford protein method, and 20 μg of proteins for each sample were separated by SDS-PAGE followed by transfer to polyvinylidene difluoride (PVDF) membranes. The membranes were blocked for 1 h at room temperature with 5% nonfat milk in the TBST buffer and incubated with a specific primary antibody diluted in TBST buffer overnight at 4 °C. After extensive washes by TBST buffer, the membranes were incubated with a corresponding secondary antibody for 1 h at room temperature, and the immunoreactive bands were visualized by using an ECL kit (Vazyme Biotech Co. Ltd., Nanjing, China). The original pictures of all Western blot experiments are shown in Figure S6.

### 2.7. GST Pull-Down Assay

The GST fusion protein expressed in *E. coli* was lysed with the binding buffer (20 mM Tris⋅HCl, pH 7.5, 150 mM NaCl, 0.1% Nonidet P-40, 1 mM dithiothreitol, 10% glycerol, 1 mM EDTA, 2.5 mM MgCl_2_, and 1 µg/mL leupeptin) for 30 min at 4 °C. Glutathione-Sepharose beads (Thermo Fisher Scientific) were incubated with bacterial lysates containing expressed recombinant GST-EZH2 mutant proteins for 4 h at 4 °C. After extensive washing of the beads, 1.0 µg of purified His×6-YY1 was added, and the samples were incubated for another 4 h. The washed beads with bound proteins were resuspended in an SDS-containing sample buffer and analyzed by SDS-PAGE.

### 2.8. Immunostaining Assay

To detect endogenous protein subcellular localization, cells were seeded in a 12-well plate with aseptic glass coverslips overnight. After PBS washes, the cells were fixed in 4% paraformaldehyde (PFA) for 10 min, permeabilized with 0.1% Triton-X 100 in PBS for 10 min at room temperature, blocked with 1% BSA for 1 h at 37 °C, and incubated with a primary antibody in 0.1% BSA overnight at 4 °C. The cells were then washed thrice with PBS and incubated with a secondary antibody for 45 min at room temperature followed by PBS washes and DAPI staining. To determine subcellular localization of peptides, the overnight cultured cells on coverslips were treated by 30 µM of N-terminal fluorescein isothiocyanate (FITC)-labeled peptides for 48 h. After the steps of PBS washes, fixation by 4% PFA, and permeabilization by 0.1% Triton-X 100, the cells were then washed thrice with PBS and stained by DAPI. Images of cells on coverslips were taken using a Delta-Vision Elite (GE, Boston, MA, USA).

### 2.9. Cell Viability Assay

Cell proliferation was determined using WST-1 assays. Briefly, cells were seeded into a 96-well plate at a density of 3 × 10^3^ cells/well in triplicate, cultured overnight, and then treated by different concentrations of peptides, as well as a vehicle control. After 48 h, 10 μL of WST-1 solution (Roche, Indianapolis, IN, USA) was added to each well followed by an additional 4 h of incubation and measurement of absorbance at 450 nm using a microplate reader. Cell viability of each treatment was determined by its OD_450nm_ percentage against that of the vehicle control. An IC_50_ (the half maximal inhibitory concentration) of a peptide represented the concentration at which it achieved killing of half the total number of cells within 48 h and was calculated using the GraphPad Prism 5.0 software. In cotreatments of peptides and doxorubicin (DOX), a PBS control or each peptide at a concentration of its IC_50_ value was used together with DOX at different concentrations (5, 10, 15, 20, 25, and 30 nM). After 48 h, cell viability and IC_50_ values of DOX in the cotreatment were determined as described above.

### 2.10. Wound Healing Assay

Cells were cultured to confluence and treated by 30 µM of peptide for 24 h prior to creating scratch wounds in the plates using a sterile pipette tip. The wounds were imaged at the time points of 0 h (creating the scratches) and 48 h. The migration rates were quantified based on scratched area measurement at the two time points.

### 2.11. Cell Apoptosis Assay

Cells cultured overnight in 12-well plates were individually treated with PBS, 30 μM of a control (Cont) peptide, YPB and OPB peptides (without FITC labeling), as well as 15 µM of tamoxifen, for 48 h. The cells were collected by trypsinization and washed twice by ice-cold PBS, followed by Annexin V-FITC and propidium iodide (PI) staining for 10 min using the reagents in an Annexin V-FITC Apoptosis Detection Kit (Vazyme Biotech Co. Ltd., Nanjing, China). The ratios of apoptotic cells were evaluated using flow cytometry (AccuriC6, BD Biosciences, San Jose, CA, USA).

### 2.12. Mouse Xenograft Study

The study was conducted according to the guidelines of the Declaration of Helsinki, and approved by the Institutional Review Board (or Ethics Committee) of Northeast Forestry University (protocol code SYXK2015002 25 January 2019). Six-week-old female athymic nude mice were injected using a 24-gauge needle with 2 × 10^6^ MDA-MB-231 cells in 0.2 mL of serum-free RPMI 1640 medium mixed with Matrigel (BD Biosciences) at a 1:1 ratio; thus, 100 μL of cells mixed with 100 μL of Matrigel were subcutaneously injected into the right or left flank of each nude mouse purchased from Vital River Laboratories (Beijing, China). Mice were randomly separated into 3 groups of 7 mice per group. After the xenograft tumors reached a volume around 100 mm^3^ (V = a × b^2^/2, where a and b represent the length and width, respectively), the YPB, OPB, and Cont peptides (30 μM in 100 μL) were intratumorally injected into the tumors of the mice of the 3 groups every other day for 22 days. Three days after the last injection, the mice were sacrificed, and tumors were excised and weighed. Immunohistochemical studies were carried out to analyze the xenograft tissues using different antibodies, and the staining intensity was quantified by the ImageJ software.

### 2.13. Immunohistochemistry

Tumor tissues were fixed in 4% paraformaldehyde at room temperature, dehydrated by an increasing gradient of ethanol solutions, embedded in optimum cutting temperature compound (OTC), and sectioned into 5 μm slices. The sections were individually incubated at 4 °C overnight with specific primary antibodies against Ki-67, caspase 3, cleaved caspase 3, and cleaved PARP. After extensive wash, the sections were incubated with biotinylated secondary antibodies at room temperature for 1 h followed by diaminobenzidine staining and hematoxylin counter staining. Images were captured using the M8 Microscope and Scanner (PreciPoint, Freising, Germany).

### 2.14. ChIP-Seq Library Preparation and Sequencing

The ChIP-seq libraries were prepared using the NEBNext Ultra II DNA Library Prep Kit for Illumina (cat# E7645S) from New England Biolabs Inc. (Ipswich, MA, USA). The following steps were followed for library preparation: end repair, 5′ phosphorylation, dA-tailing, adapter ligation, U excision, cleanup of adaptor-ligated DNA without size selection, and library amplification for 12 to 13 cycles followed by clean-up with Agencourt AMPure XP. The quality of the libraries was assessed using the Agilent High Sensitivity DNA kit (cat# 5067-4626) on the Agilent 2100 Bioanalyzer (Agilent Technologies, Santa Clara, CA, USA) and quantified using the NEBNext Library Quant kit for Illumina (cat# E7630S) from New England Biolabs Inc. The libraries were sequenced using the NextSeq 500 High Output v2 kit (75 cycles) (cat# FC-404-2005) on the NextSeq 500 platform from Illumina (San Diego, CA, USA). IGV was used to generate browser tracks [36].

### 2.15. Statistical Analysis

Experiments were carried out at least 3 times unless otherwise stated. The software GraphPad Prism 5.0 was used for statistical analysis and the data are shown as mean ± S.D. Student’s t-test and one way ANOVA were employed to assess the statistical significance of differences between data sets. A *p*-value of lower than 0.05 was considered to be significant. *p* < 0.05 (*), *p* < 0.01 (**), and *p* < 0.001 (***)

## 3. Results

### 3.1. YY1 Associates with EZH2 in Breast Cancer Cells

**Figure 1:**
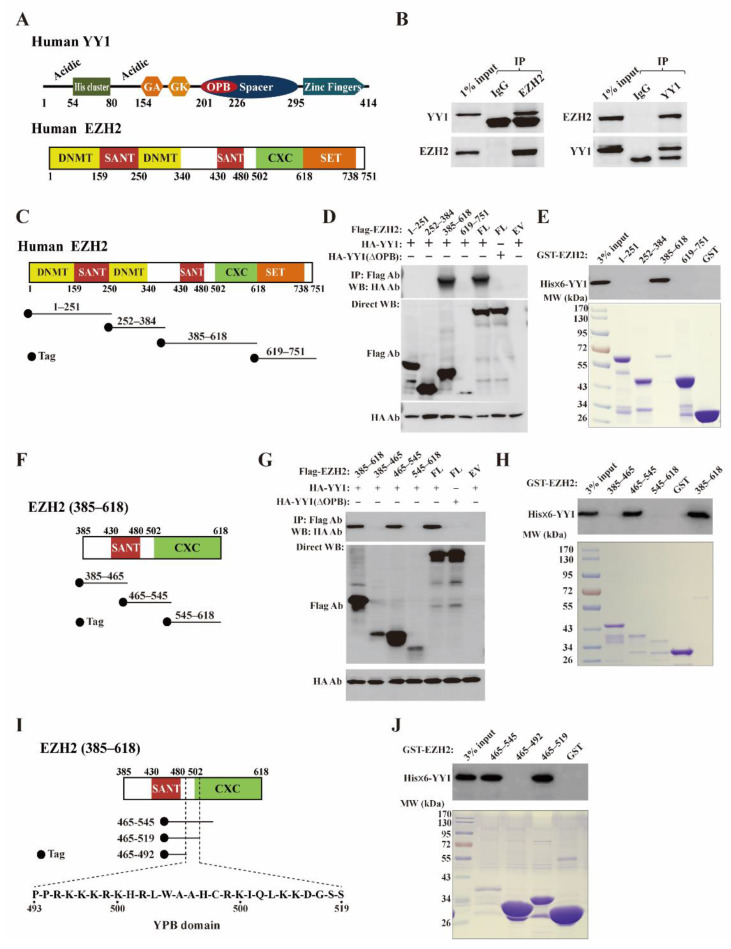
Identification of YY1 binding site on EZH2 protein. (**A**) Schematic diagrams of the domain structures of YY1 and EZH2 proteins based on previous reports [30,39]. In the domain structure of YY1, “Acidic” represents a region containing enriched aspartic and glutamic acids, “His cluster” contains 11 consecutive histidines, “GA” and “GK” depict regions enriched by glycine/alanine and glycine/lysine, respectively; OPB: oncoprotein binding; the Spacer region and Zinc finger domain are also denoted. In the domain structure of EZH2, “DNMT” is the binding domain of DNA methyltransferase 1 (DNMT1), “SANT” denotes the SANT domain regulating chromatin accessibility [40],“CXC” is the cysteine-rich domain, and the SET domain is also denoted. (**B**) Western blot analyses of endogenous YY1 and EZH2 interaction by co-immunoprecipitation (co-IP). MDA-MB-231 cell lysates (800 µg) were incubated with 2 µg of EZH2 antibody (cat# 5246S, left) or YY1 antibody (cat# sc-281, right), followed by the incubation of Protein A/G beads and extensive washing. The samples were analyzed by Western blot using EZH2 and YY1 antibodies as indicated. (**C**,**F**,**I**) Diagram of EZH2 mutants generated to map its YY1 binding region. The tag is either an HA epitope or GST. (**D**,**E**,**G**–**I**) Co-IP (**D**,**G**) and GST-pull down (**E**,**H**,**J**) studies to determine the YY1 binding region on the EZH2 protein. FL: full length; EV: empty vector; IP: immunoprecipitation; Direct WB: Western blot analysis of samples without IP; YPB: YY1 protein binding.

Both YY1 and EZH2 have several functional domains responsible for their various biological activities (Figure 1A). As a master transcription factor regulating different biological processes, YY1 recruits PcG proteins to mediate gene silencing [16]. Importantly, YY1 was reported to directly bind EZH2 [30]. However, the precise binding region of YY1 on the EZH2 protein has never been elucidated. Thus, we first carried out reciprocal co-immunoprecipitation (co-IP) using the lysates of MDA-MB-231 cells, and observed the association of endogenous YY1 and EZH2 (Figure 1B). To map the YY1 binding region on EZH2, we generated vectors expressing different EZH2 regions (1–251), (252–384), (385–618), and (619–751) with an N-terminal Flag tag (Figure 1C), and used them in co-IP studies with a plasmid pcDNA3/HA-YY1 expressing HA-YY1. As shown in Figure 1D, Flag-EZH2 wt and its mutant (385–618), but not the others, could bring down HA-YY1. Consistently, in vitro binding assays also demonstrated that GST-EZH2 (385–618) could interact with His×6-YY1 (Figure 1E), suggesting that the YY1 binding site resided in the residues 385–618 of EZH2. Through surface plasmon resonance (SPR) analysis using the Biacore (GE), we confirmed the binding of this region with YY1 with purified GST as a negative control, and determined the dissociation equilibrium constant (KD) as 2.7 × 10^−7^ (Figure S1A–E). Previous studies mapped the EZH2 binding site on YY1, named the OPB domain (also called REPO) [37]. As predicted, wt EZH2 did not show detectable binding affinity to HA-YY1 (ΔOPB), a mutant with the OPB domain deleted, in the co-IP experiments (Figure 1D).

Next, we generated three additional Flag-tagged EZH2 mutants (385–465), (465–545), and (545–618) (Figure 1F). In the co-IP studies, we observed that the EZH2 (465–545) mutant, but not the other two, could interact with HA-YY1 (Figure 1G). Again, HA-YY1 (ΔOPB) that did not bind EZH2 was used as a negative control. In line with these data, GST-EZH2 (465–545) mutant could also pull down His×6-YY1 (Figure 1H), indicating that this region was responsible for YY1 interaction. When tested by Biacore, the KD value of the binding between GST-EZH2 (465–545) and His×6-YY1 was determined as 5.3 × 10^−7^ (Figure S1F–H).

To further narrow down the YY1 binding site, we generated two shorter GST-EZH2 mutants, (465–519) and (465–492), based on GST-EZH2 (465–545) (Figure 1I). Strikingly, GST-EZH2 (465–519), but not the (465–492), could interact with His×6-YY1, suggesting that the YY1 binding site was likely located in the stretch of residues 493–519 of EZH2 (Figure 1J). We thus named this region of EZH2 the YY1 protein binding (YPB) domain that contains 27 residues between residues 493 and 519 (Figure 1I).

**Figure 2:**
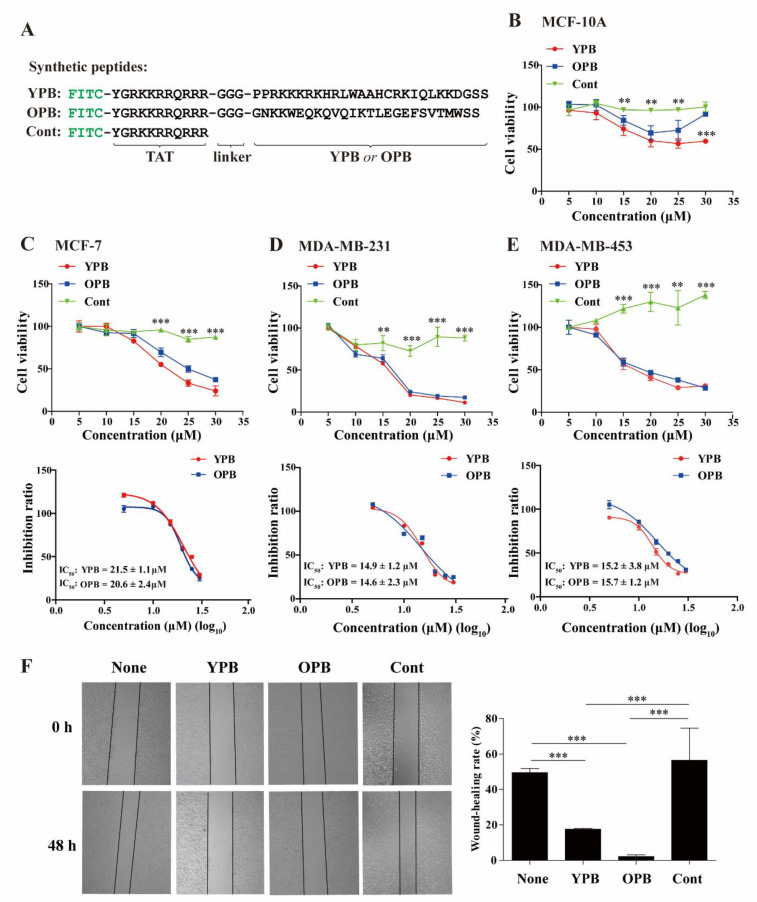
YPB and OPB peptides reduced breast cancer cell viability and disrupted YY1-EZH2 interaction**.** (**A**) The design and sequences of the synthetic YPB, OPB, and Cont peptides. FITC: fluorescein isothiocyanate; TAT: a cell-penetrating peptide derived from human immunodeficiency virus. (**B**–**E**) Inhibitory effects the YPB and OPB peptides on breast cancer cells. MCF-10A, MCF-7, MDA-MB-231, and MDA-MB-453 cells were individually treated with different concentrations of YPB, OPB, and Cont peptides for 48 h, followed by WST-1 assays to determine cell proliferation. Then, cell viability (upper panels) and peptide inhibition ratios (lower panels) were calculated, with the IC_50_ values in these cell lines embedded in the graphs. (**F**) Scratch assay to test the effects of PBS, YPB, OPB, and Cont peptides on cell migration. Scratches on the plates with overnight cultured MDA-MB-231 cells were created with immediate addition of PBS, 30 µM of YPB, OPB, and Cont peptides into the medium. The scratches were imaged after 48 h, and the quantitation of cell migration was presented at right. Viability of nontumorigenic MCF-10A cells treated by the 3 peptides. Data represent the mean ± S.D. ** *p* < 0.01, *** *p* < 0.001.

We synthesized a fusion peptide, in which the N-terminus of a synthetic YPB domain was fused to a cell-penetrating peptide derived from the transactivator of transcription (TAT) of human immunodeficiency virus and could facilitate cross-membrane transport of the peptide [38]. The two parts were linked by three glycines (3G), and fluorescein isothiocyanate (FITC) was conjugated to the fusion peptide’s N-terminus (Figure 2A). When tested by Biacore, this synthetic FITC-TAT-3×Glycine-YPB peptide (hereafter designated as the YPB peptide) could interact with His×6-YY1 with a KD of 1.6 × 10^−7^ (Figure S1I), suggesting that the YPB domain is sufficient to bind YY1.

### 3.2. YPB and OPB Peptides Reduced Breast Cancer Cell Viability and Migration

Previous studies indicated that EZH2 was recruited by YY1 to the promoters of target genes [41]. We reported that the peptide derived from the OPB sequence could block YY1 binding to several oncoproteins [30,42]. Thus, to evaluate the activity of the YPB peptide, we also synthesized the FITC-TAT-3×Glycine-OPB peptide (hereafter designated as the OPB peptide) and an unfused FITC-TAT peptide (designated as the Cont peptide) as positive and negative controls, respectively (Figure 2A).

To test the effects of the YPB peptide in cell-based assays, we used a series of peptide concentrations to treat different breast cancer cell lines, including MCF-7 and two triple negative breast cancer (TNBC) cell lines, (MDA-MB-231 and MDA-MB-453), as well as nontumorigenic MCF-10A cells. After 48 h of the treatment, we determined cell proliferation by the WST-1 assay and calculated cell viability and inhibition ratios. When testing MCF-10A cells, YPB and OPB peptides showed modest inhibition, and their IC_50_ could not be calculated because 50% inhibition was not reached with continuously increased peptide concentrations (Figure 2B). In the three breast cancer cell lines, both YPB and OPB peptides, but not Cont, could remarkably reduce the viability and thus exhibited significant inhibition ratios in a dose-dependent manner, while the Cont peptide lacked any detectable inhibition to these cells (Figure 2C–E). After calculating the inhibition rates, the YPB and OPB showed comparable IC_50_ values around 20 μM among the three breast cancer cell lines (embedded in Figure 2C–E). We also tested the effects of the two peptides on the migration of MDA-MB-231 cells and observed that both YPB and OPB peptides could markedly reduce cell migration compared to the control groups (Figure 2F). MCF-7 and MDA-MB-453 cells showed very similar migration tendency to MDA-MB-231 cells when treated by YPB and OPB peptides (data not shown).

### 3.3. The YPB and OPB Peptides Induced Breast Cancer Cell Apoptosis

**Figure 3:**
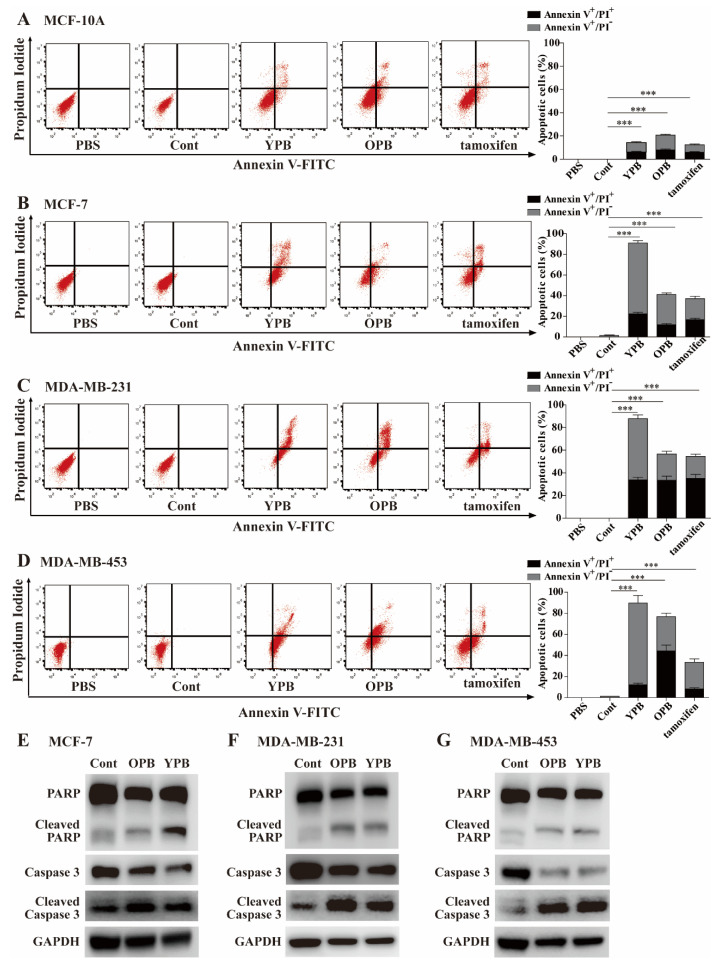
Evaluation of apoptosis in breast cancer cells treated by the peptides**.** (**A**–**C**) Flow cytometric analyses of breast cancer cells treated by the peptides. MCF-10A (**A**), MCF-7 (**B**), MDA-MB-231 (**C**), and MDA-MB-453 (**D**) cells were individually treated by PBS, 30 µM of Cont, YPB, and OPB peptides (unlabeled), and 15 µM of tamoxifen for 48 h, followed by the staining of Annexin V-FITC and PI. The apoptotic rates of the treated cells were analyzed by flow cytometry with representative images shown at left and quantitative apoptotic rates calculated by FlowJo software shown at right. Data represent the mean ± S.D. * *p* < 0.05, ** *p* < 0.01, *** *p* < 0.001. (**E**–**G**) Western blot analyses of apoptotic markers in breast cancer cells treated by the peptides. MCF-7 (**E**), MDA-MB-231 (**F**), and MDA-MB-453 (**G**) cells were treated as described in (**A**–**C**). The cell lysates were subjected to Western blot analyses using indicated antibodies with GAPDH as a loading control.

To evaluate potential reasons for reduced cell viability, we treated the mammary cells individually with the vehicle (PBS), Cont, YPB, and OPB peptides (without FITC labeling), as well as tamoxifen as a positive control, followed by the staining of Annexin V-FITC and PI to identify early and late apoptotic cells, respectively. The analyses by a fluorescence activated cell sorter (FACS) showed that tamoxifen, YPB, and OPB peptides could significantly increase the pro-apoptotic portions of breast cancer cells compared to the Cont-treated cells, while the PBS treatment did not lead to any apoptotic population (Figure 3A–D). Compared to breast cancer cells, MCF-10A cells exhibited much-reduced apoptotic response to YPB and OPB peptides. As a result, the apoptotic rates of the two peptides were determined as YPB: 14.23 ± 1.34% and OPB: 20.79 ± 1.94% in MCF-10A cells; YPB: 91 ± 3.65% and OPB: 41.1 ± 2.7% in MCF-7 cells; YPB: 87.8 ± 5.75% and OPB: 56.5 ± 5.5% in MDB-MB-231 cells; YPB: 89.8 ± 8.7% and OPB: 76.9 ± 8.75% in MDA-MB-453 cells. Therefore, YPB and OPB peptides showed much-reduced activity in inducing cell apoptosis compared to that in breast cancer cells, in line with the cell viability data in Figure 2B–E. Consistently, YPB and OPB peptides could increase the cleavage of PARP and caspase 3 in these breast cancer cell lines when evaluated by Western blot analysis (Figure 3E–G), validating their pro-apoptotic activity in these cells.

We also tested whether the cotreatments of YPB and OPB peptides with DOX could improve the inhibition of breast cancer cell proliferation. As shown in Figure S2A–C, the presence of YPB and OPB peptides could markedly reduce the IC50 of DOX in MDA-MB-231 cells, suggesting potential clinical applications of the two peptides.

### 3.4. The YPB and OPB Peptides Disrupted YY1-EZH2 Interaction and Mostly Stayed in Nuclei

**Figure 4:**
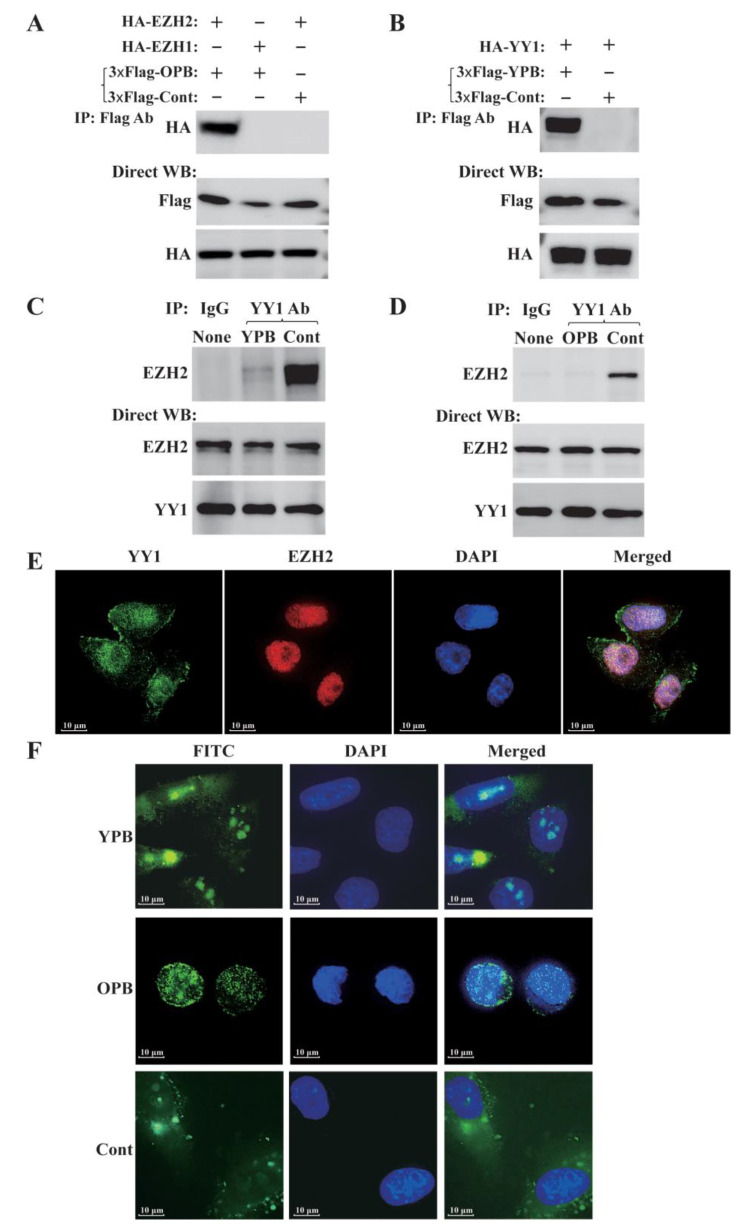
Examination of YPB and OPB peptides in blocking YY1-EZH2 interaction, and their subcellular localization. (**A**,**B**) Co-IP experiments to determine OPB and YPB interaction with EZH2 and YY1, respectively. In A, 3×Flag-OPB-2A1-EGFP, 3×Flag-Cont-2A1-EGFP expression vectors, and an empty vector were individually cotransfected with HA-EZH2, HA-EZH1 expression vectors, or an empty vector. Cell lysates were co-IPed using Flag antibody followed by Western blot analysis using HA antibody. In B, similar to A but using 3×Flag-YPB-2A1-EGFP and HA-YY1 in the transfection and co-IP studies. (**C**,**D**) Examination of YPB and OPB peptides’ effects on YY1-EZH2 interaction. MDA-MB-231 cell lysates were treated by 30 μM of Cont, OPB, and YPB peptides for 4 h, followed by co-IP using 2 µg of normal IgG, YY1, and EZH2 antibodies. The co-IPed samples were analyzed by Western blot using YY1 and EZH2 antibodies to evaluate the effects of YPB (**C**) and OPB (**D**) peptides on YY1-EZH2 interaction. (**E**) Examination of YY1 and EZH2 colocalization in cells. MDA-MB-231 cells were immunostained by YY1 and EZH2 antibodies. DAPI was used to visualize nuclei. (**F**) Detection of subcellular localization of peptides. MDA-MB-231 cells were treated by 30 μM of Cont, OPB, and YPB peptides for 48 h, followed by DAPI staining. The peptides were all N-terminal FITC-labeled and thus could emit green fluorescence.

To test whether YPB and OPB peptides could bind YY1 and EZH2, respectively, we generated three expression vectors. The vector 3×Flag-YPB-2A1-EGFP contained the 2A1 sequence in mRNA, which could cause “ribosomal skipping” [32] to produce separate YPB and EGFP products. The vectors 3×Flag-OPB-2A1-EGFP and 3×Flag-Cont-2A1-EGFP followed the same principle. In the co-IP studies, 3×Flag-OPB, but not 3×Flag-Cont, could bind HA-EZH2, and this binding affinity could not be extended to EZH1, an EZH2 homolog (Figure 4A). Similarly, 3×Flag-YPB, but not 3×Flag-Cont, could also bind to HA-YY1 (Figure 4B).

### 3.5. The YPB and OPB Peptides Inhibited Xenograft Tumor Growth in Nude Mice

**Figure 5:**
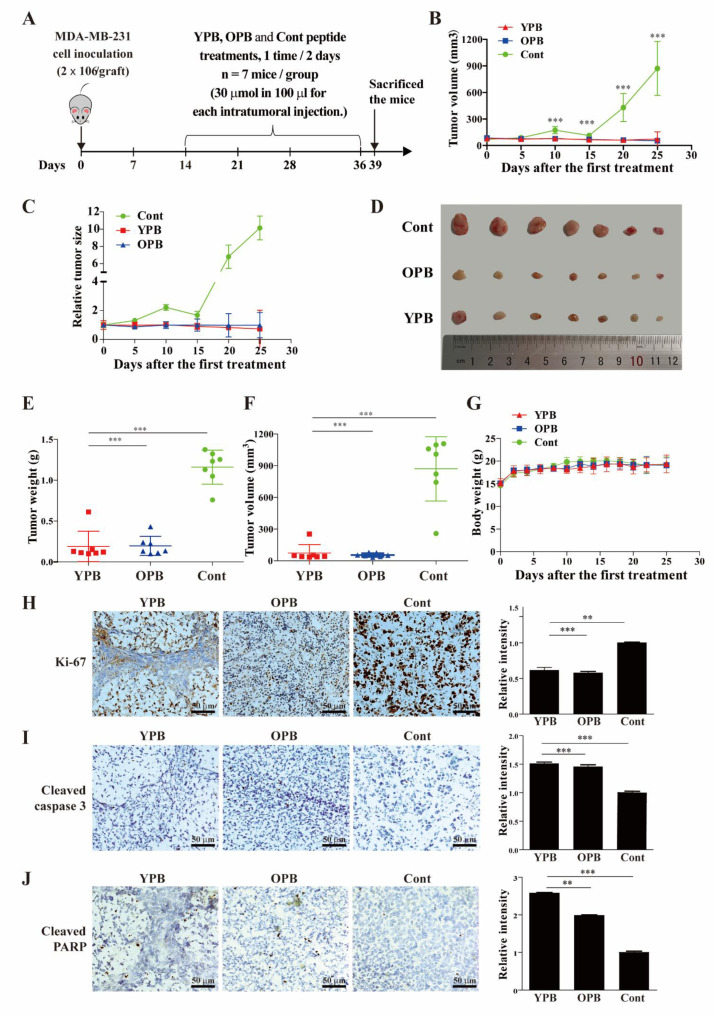
Effects of the peptides on breast cancer growth in a xenograft mouse model**.** (**A**) The experimental design of the mouse xenograft study. MB-MDA-231 cells (2 × 10^6^) were subcutaneously inoculated at the right or left flank of each BALB/c nude mouse. After the tumors developed to a volume of approximate 60–100 mm^3^, the mice were randomly divided into 3 groups with 7 mice in each group. The YPB, OPB, and Cont peptides were intratumorally injected into the tumors of the mice in the three groups correspondingly with a dosage of 30 μM in 100 µL per injection and injected every other day for 22 days. The mice were sacrificed 3 days after the last injection. (**B**,**C**) Tumor volumes (**B**) and their relative sizes (**C**) from the mice in the 3 groups after the initial peptide injection. (**D**) Image of actual xenograft tumors excised from the mice treated by the peptides. (**E**,**F**) The weights and volumes of the excised tumors after the mice were sacrificed. (**G**) The body weights of the mice in the 3 groups after the initial peptide injections. (**H**–**J**) Immunohistochemical analyses of xenograft tumors. Representative images of immunohistochemical staining using antibodies against Ki-67 (**H**), cleaved caspase3 (**I**), and cleaved PARP (**J**) are presented in the left panels, and their quantification was shown in the right panels. The quantification was carried out under 400× magnification in 3 randomly selected areas in each tumor, and the data shown are presented as mean ± SD of 3–5 tumor samples from individual mice in each group. Values represent the mean ± S.D., ** *p* < 0.01, *** *p* < 0.001.

We next tested whether the inhibition of breast cancer cells by YPB and OPB peptides could be extended to an in vivo model. When the xenograft tumors formed by subcutaneously inoculated MDA-MB-231 cells in the flanks of BALB/c-(nu/nu) female mice reached about 100 cm^3^, the mice were randomly divided into three groups with seven mice in each group. YPB, OPB and Cont peptides (30 μM in 100 µL) were intratumorally injected into the mice of the three groups every other day for 22 days (Figure 5A). Three days after last injection, the mice were sacrificed, and the excised tumors were weighed and analyzed by immunohistochemistry.

The tumors in the groups treated by the YPB and OPB peptides started to show significantly reduced growth rates compared to the group of the Cont peptide at 10 days after peptide injection, which were maintained throughout the course of the experiment (Figure 5B). Actually, when looking at the relative tumor sizes, OPB peptide stopped tumor growth, while YPB peptide could even cause partial tumor remission (Figure 5C). After sacrificing the mice, the excised tumors (Figure 5D) from the YPB and OPB groups showed significantly reduced weights and volumes compared to those of the Cont group (Figure 5E,F). Notably, the average body weights of the three treatment groups did not show significant difference, pointing to a lack of significant side effects by the YPB and OPB treatments (Figure 5G).

In the immunohistochemical analyses, compared to the control group with Cont, the tumors treated by the YPB and OPB peptides exhibited a markedly decreased Ki-67 signal, suggesting reduced cell proliferation (Figure 5H). Consistent with the in vitro observation, YPB and OPB peptides also significantly increased the intensity of both cleaved caspase 3 and cleaved PARP in the tumor samples (Figure 5I,J), indicative of their pro-apoptotic activity in the xenograft tumors.

### 3.6. ChIP-Seq Studies Revealed the PTENP1 Gene as a Primary Target of YPB and OPB Peptides

YY1 recruited EZH2 to mediate gene expression. Thus, disruption of this regulation by either YPB or OPB peptide may contribute to the retarded cell proliferation and reduced tumor formation observed by us. The key role of EZH2 in epigenetic regulation is its activity as a histone methyltransferase to promote H3K27me3, which is a hallmark of gene repression [43]. To investigate how the YPB and OPB peptides could alter EZH2-mediated histone modification, we used an antibody against H3K27me3 to carry out ChIP-seq analysis in MDA-MB-231 cells treated by the three peptides. The ChIP-seq dataset of this study is available in the Gene Expression Omnibus (GEO) with an access number of GSE171954.

**Figure 6:**
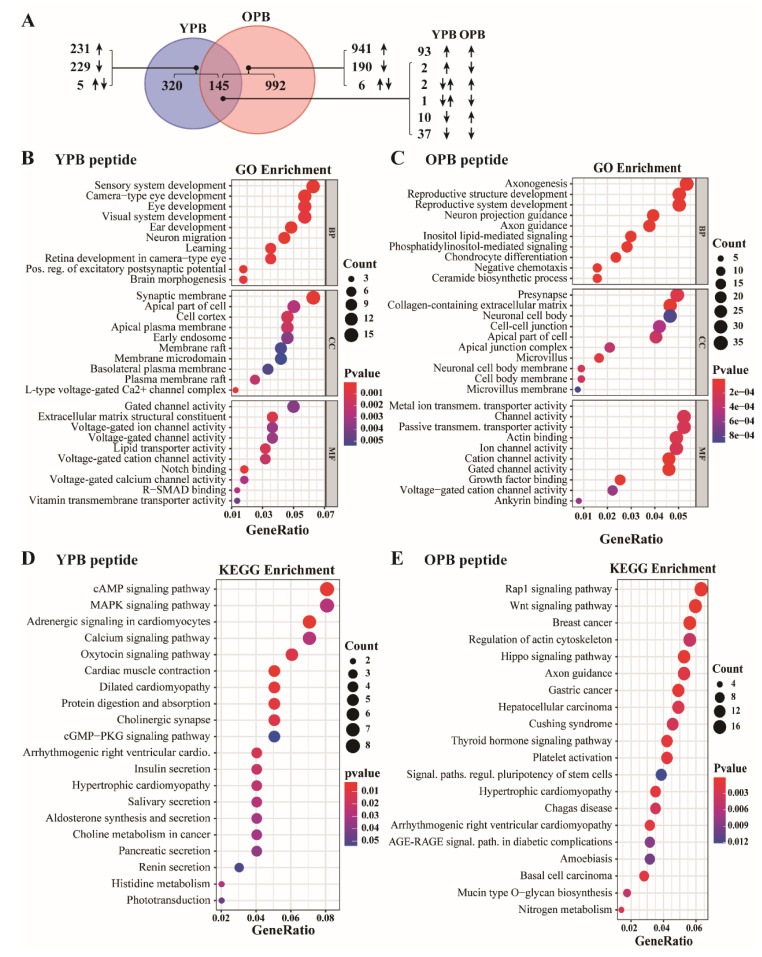
Analyses of the H3K27me3 ChIP-seq data in peptide-treated MDA-MB-231 cells. (**A**) Numbers of genes with altered H3K27me3 ChIP-seq signal in the vicinity (−1000 to +1000 bps) of their TSSs when treated by YPB and OPB peptides, and their overlapped genes. The false discovery rate (FDR) was set as <0.05 and the threshold for the fold of change (FC) of H3K27me3 signal was 1.5. The upward and downward arrows designate the genes with increased or decreased H3K27me3 signal, respectively. The genes with both upward and downward arrows indicate that the genes have both increased and decreased H3K27me3 signal in their vicinity. (**B,C**) The top 10 GO terms of the GO analyses of the ChIP-seq data in MDA-MB-231 cells treated by YPB (**B**) and OPB (**C**) peptides. The analyses included biological process (BP), cell component (CC), and molecular function (MF) categories. (**D**,**E**) KEGG pathway enrichment analyses of the ChIP-seq data in MDA-MB-231 cells treated by YPB (**D**) and OPB (**E**) peptides.

We focused on identifying altered H3K27me3 signal (or peaks) in the vicinity (−1000 to +1000 base pairs, or bps) of the transcriptional start sites (TSSs) of the annotated genes between YPB/OPB- and Cont-treated samples. In these analyses, we set the false discovery rate (FDR) as <0.05 and the threshold for the fold of change (FC) of H3K27me3 signal as 1.5. With these settings, we detected that, between the YPB peptide-treated and Cont peptide-treated cells, 475 regions, corresponding to 465 genes, showed differential H3K27me3 signal. Among them, 231, 229, and 5 genes showed increased, decreased, or both increased and decreased H3K27me3 signal, respectively (Figure 6A). Similarly, among the samples treated by the OPB peptide, we identified 1207 regions, corresponding to 1137 genes, with differential H3K27me3 signal compared to the Cont-treated samples. Among them, 941, 190, and 6 genes exhibited increased, decreased, or both increased and decreased H3K27me3 signal, respectively, compared to the control samples (Figure 6A). When comparing the data of YPB and OPB peptide treated cells, we identified 145 genes that demonstrated altered H3K27me3 signal in both treatments. Among them, 93 and 37 genes showed consistent increase or decrease of H3K27me3 signal, respectively, compared to the Cont-treated controls; the other 15 exhibited differential H3K27m3 signal changes (Figure 6A).

We employed the comprehensive Gene Ontology (GO) enrichment to analyze the functions and associated biological processes of the genes with differential H3K27me3 signal in MDA-MB-231 cells treated by YPB and OPB peptides versus Cont (Figure 6B,C). The analyses included biological process (BP), cell component (CC), and molecular function (MF) categories. We also used the Kyoto Encyclopedia of Genes and Genomes (KEGG) pathway enrichment method to analyze these genes and determine the relevant signaling pathways (Figure 6C,D). In the samples treated by both YPB and OPB peptides, the GO analyses revealed that the most enriched genes were those involved in neural cell development and transmembrane transport (Figure 6B,C, Table 1). In the KEGG pathway analyses, YPB treatment primarily altered genes involved in the cAMP and MAPK pathways, while OPB treatment impacted RAP1, WNT, and HIPPO pathways, as well as signaling pathways in breast, gastric, and liver cancers (Figure 6D,E, Table 1).

At the time of preparing the manuscript, there were eight YY1 and two EZH2 ChIP-seq datasets available in the ENCODE and GEO databases, but none of them used mammary cells [44]. To understand YY1- and EZH2-regulated signaling pathways, we carried out integrative KEGG analyses of these eight and two datasets, respectively (Figure S3A,B). When comparing these integrative analysis results with our dataset, we found that both YPB and OPB ChIP-seq data showed several overlapping signaling pathways with the those from the EZH2-regulated pathways, but no overlap with those of YY1-regulated pathways (Figure S3).

**Figure 7:**
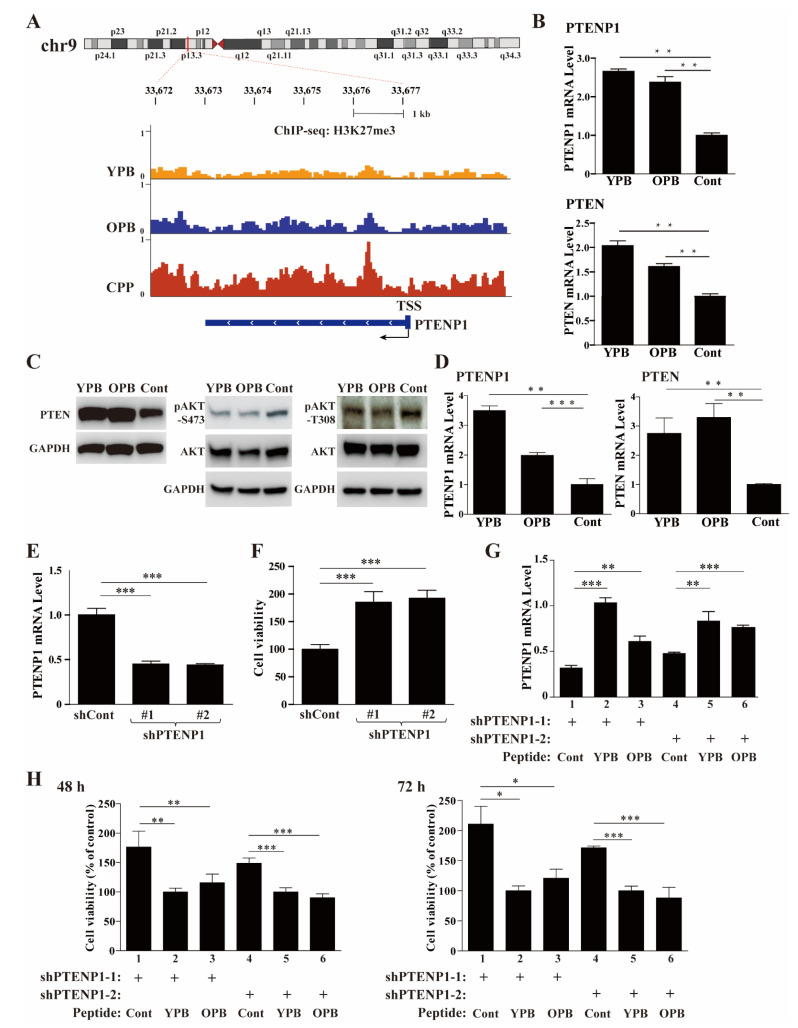
PTENP1 is one of the primary targets of YPB and OPB peptides in breast cancer cells. (**A**) Gene browser tracks of the H3K27me3 ChIP-seq at the PTENP1 locus on chromosome 9 (chr9) in the genomic DNA of MDA-MB-231 cells treated by the peptides. The yellow, blue, and red peaks represent the degrees of H3K27me3 enrichment after the treatments for 48 h by 30 µM of the YPB, OPB, and Cont peptides, respectively. The region of the PTENP1 gene is labeled at the bottom panel. TSS: transcription start site. (**B**,**C**) Alterations of genes in the AKT pathway in response to the peptide treatments. After the treatment of MDA-MB-231 cells by the YPB, OPB, and Cont peptides, the cell lysates were analyzed by RT-qPCR to evaluate PTENP1 and PTEN transcript levels (**B**), and by Western blot analysis to determine the levels of PTEN, pAKT-S473, p-AKT-S308, and AKT, with GAPDH as a control (**C**). (**D**) Examination of PTENP1 and PTEN expression in xenograft tumors by qPCR. (**E**,**F**) Effects of shPTENP1-1 and -2 on the expression of the endogenous PTENP1 transcript (**E**) and viability (**F**) of MDA-MB-231 cells. (**G**,**H**) Effects of the cotreatments by shPTENP1-1/2 and each of the peptides on the PTENP1 transcript levels (**G**) and the viability (**H**) of MDA-MB-231 cells. The cells were infected by lentiviruses carrying either of the shPTENP1-1/2, and then treated for 48 h by 30 µM of the YPB, OPB, and Cont peptides. Relative PTENP1 transcript levels were determined by RT-qPCR, while the cell viability was calculated based on WST-1 assays. All data were normalized against the data of YPB. Each experiment was repeated at least 3 times with representative data presented. Data are shown as the mean ± S.D. * *p* < 0.05, ** *p* < 0.01, *** *p* < 0.001.

As we predicted that YPB and OPB could disrupt EZH2 recruitment by YY1, we checked the overlapped genes with reduced H3K27me3 signal in response to YPB and OPB treatments. Among these 38 genes (Figure 6A and Table S1), we discovered that PTENP1, the PTEN pseudogene, showed markedly reduced H3K27me3 signal (Figure 7A). The PTENP1 gene transcribes a lncRNA that acts as a competitive endogenous RNA (ceRNA) to improve PTEN mRNA stability and consequently promote PTEN expression [45]. Consistently, quantitative PCR (qPCR) analyses showed that both PTENP1 and PTEN transcripts were upregulated in MDA-MB-231 cells treated by YPB and OPB peptides versus Cont (Figure 7B). As a phosphatase, PTEN converts PIP3 to PIP2, which reduces AKT binding to the plasma membrane and consequently decreases its activation. Indeed, with increased PTEN protein expression in MDA-MB-231 cells treated by YPB and OPB compared to Cont, we also detected simultaneously reduced AKT phosphorylation at S473 and T308 (Figure 7C). Consistently, the xenograft samples treated by YPB and OPB peptides also showed increased PTENP1 and PTEN transcript levels compared to those treated by Cont peptide (Figure 7D). Furthermore, shRNA-mediated knockdown of YY1 and EZH2 could also significantly increase the expression of both PTENP1 and PTEN genes in MDA-MB-231 cells (Figure S4A,B).

Next, we tested whether YPB- and OPB-mediated PTENP1 upregulation played a crucial role in reduced proliferation of breast cancer cells. We generated two shRNAs specifically targeting PTENP1 (shPTENP1-1 and shPTEN-2) and a control shRNA (shCont), and produced lentiviruses carrying the expression cassettes of these shRNAs. The introduction of the PTENP1 shRNAs into MDA-MB-231 cells through lentiviral infection could significantly reduce PTENP1 levels with concurrently enhanced cell viability compared to the cells expressing shCont (Figure 7E,F). Next, we individually used the peptides to treat the cells expressing the shPTENP1-1 and shPTEN-2, and observed partially restored PTENP1 expression caused by the YPB and OPB peptides (Figure 7G, columns 2 and 3 versus 1, columns 5 and 6 versus 4) and concomitantly reduced cell proliferation when compared to the Cont treatment at both 48 h and 72 h time points (Figure 7H, columns 2 and 3 versus 1, columns 5 and 6 versus 4). The results indicated that YPB and OPB peptides could promote PTENP1 expression to inhibit MDA-MB-231 cell proliferation. In addition to PTENP1, we also identified several other genes that showed both EZH2 enrichment (based on two reported datasets, ENCFF545XSF and ENCFF521AKL, from the ENCODE database) and H3K27me3 changes (either increase or decreases) when treated by YPB and OPB peptides versus Cont peptide (Figure S5A–C). The H3K27me3 alterations of these genes were generally less profound than that of PTENP1, and their contributions to the anticancer activities of YPB and OPB peptides need future investigation.

**Figure 8:**
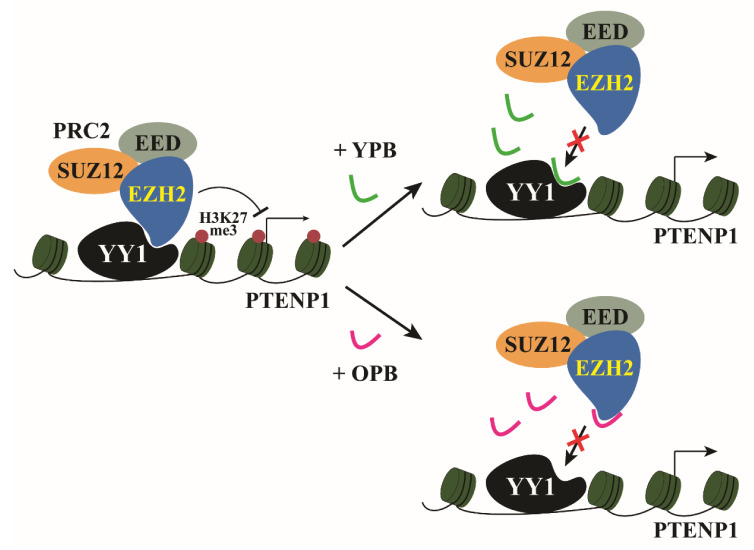
Schematic models of the inhibition of PTENP1 expression by the YPB and OPB peptides. In breast cancer cells, YY1 recruits EZH2 and its associated PRC2 to mediate H3K27me3 in the PTENP1 promoter and repress its expression. YPB and OPB can bind either YY1 or EZH2, respectively, to disrupt the recruitment of EZH2 by YY1, leading to reduced H3K27me3 at the PTENP1 and its upregulation.

Overall, based on the experimental data presented above, both YPB and OPB peptides could disrupt YY1-EZH2 interaction, decrease EZH2 recruitment by YY1 to the promoters of target genes, such as PTENP1 (Figure 8), and reduce breast cancer cell proliferation and xenograft tumor formation.

## 4. Discussion

YY1 has been identified as an essential regulator of different epigenetic processes, including histone methylation, acetylation, and DNA methylation [25]. Meanwhile, YY1 also has activities independent of its transcriptional regulation, such as facilitating p53 ubiquitination and promoting AKT activation. YY1 overexpression has been reported in virtually all cancer types, and its therapeutic target potential in breast cancer has been demonstrated using both in vitro assays and animal models [46]. The OPB domain of YY1 is involved in binding to at least four oncoproteins, including EZH2, MDM2, AKT, and E1A [30]. We reported that synthetic peptides based on the OPB sequence could inhibit breast cancer cell proliferation, but their effects in a mouse model were not evaluated [30,42]. In the current study, we mapped the binding site of YY1 on EZH2 and identified the YPB domain consisting of 27 residues. In functional studies, we observed that both YPB and OPB peptides could disrupt YY1-EZH2 interaction, inhibit breast cancer cell proliferation, and reduce tumor formation, strongly suggesting the therapeutic potential of the two peptides in cancer treatments. We previously reported that both EZH2 and YY1 showed much lower expression in breast cancer cells than that in normal mammary cells [46], consistent with their important regulatory roles in mammary oncogenesis. The difference of EZH2 and YY1 expression between tumorigenic and nontumorigenic cells could explain the differential inhibitory effects of the two peptides in breast cancer cells and MCF-10A cells. Our data also suggested that the two peptides would potentially exhibit high specificity in targeting cancer cells and relatively low side effects in future clinical applications. Importantly, lack of target therapeutics and the poor prognosis of TNBC patients represent major hurdles in breast cancer therapies [47]. In our study, YPB and OPB peptides showed effective inhibition against two TNBC cell lines (MDA-MB-231 and MDA-MB-453) in vitro and xenograft tumors by MDA-MB-231 cells in vivo, suggesting that the two peptides could potentially be used as an efficient agent in the therapies of TNBC patients. Additionally, both EZH2 and YY1 have been reportedly overexpressed in most cancer types [25,48]; therefore, the YPB and OPB peptides can very likely exhibit inhibitory activities against other cancers.

The YPB domain (493–519) is located in the linker region between the second SANT domain and the cysteine-rich (CXC) domain of EZH2 [49], and contains a high ratio (11 of 27) of lysines and arginines, while the OPB domain has many lysines, serines, and threonines. These residues are potential targets of protein modifications. Although both EZH2 and YY1 could undergo many different modifications, including acetylation, methylation, and phosphorylation, none of the residues in either YPB or OPB domain have been reportedly modified [25,49]. Although protein modifications play an important role in protein-protein binding, they do not likely contribute to the YY1-EZH2 interaction, because the synthetic YPB and OPB peptides, lacking any modification, could efficiently disrupt their association. As we noticed, other groups also reported YY1-EZH2 interaction [41,50]. However, a previous report indicated that YY1 recruited the PRC2 through binding to EED, a scaffolding protein of the complex, but did not show interaction with EZH2 [51]. In the current study, we provided unambiguous data to demonstrate the direct binding between YY1 and EZH2 proteins using different approaches, including co-IP, GST-pull down, and Biacore analyses. Thus, it is possible that YY1 recruits PRC2 through different mechanisms to mediate gene expression. Based on previous studies, YY1 could bind both EED and SUZ12, core subunits of the PRC2, but the binding sites were not mapped to the OPB domain [51,52]. In addition, both EED and SUZ12 also have binding sites on the EZH2 protein, which do not overlap with the YPB domain [53]. Therefore, we predict that the YPB and OPB peptides could interfere with YY1-EZH2 interaction, but are unlikely to disrupt the integrity of the PRC2 itself.

We predict that both YPB and OPB peptides can disrupt YY1 interaction with multiple oncoproteins. The YPB peptide would presumably bind to the OPB domain of YY1 to block its interactions with EZH2, MDM2, and AKT, while the OPB peptides may bind to these oncoproteins, including EZH2, to prevent their binding to YY1, and could potentially interfere with their other functions. Thus, the treatment by either YPB or OPB peptide would compromise multiple YY1-regulated functions, including EZH2-mediated H3K27me3. Theoretically, disruption of YY1-recruited EZH2 to chromatin would likely cause a general reduction of H3K27me3, but we observed both decrease and increase of this modification in response to the peptide treatment in the ChIP-seq studies. Meanwhile, a number of genes showed opposing H3K27me3 changes between YPB and OPB peptide treatments, as shown in Figure 6A. YPB and OPB peptides contain 26 and 27 amino acids, respectively, in lengths, which should guarantee their high specificity. Nevertheless, we certainly cannot exclude any off-target effect of the two peptides, especially the expected binding of OPB peptide to MDM2 and AKT1 [26,30], which could indirectly contribute to the conflicting H3K27me3 changes by YPB and OPB peptide treatments. Importantly, these phenomena could be due to the presence of other EZH2 recruiters, such as TCF1, CDYL, and many lncRNAs [17,18,19,24], which could still mediate H3K27me3 in the presence of YPB or OPB peptides. It is still unclear whether the YPB domain overlaps with any binding site of these proteins or lncRNAs; this deserves clarification in future studies. In addition, based on Western blot analyses in Figure 4C,D, YPB and OPB treatments did not change endogenous EZH2 or YY1 protein levels in breast cancer cells. Thus, disruption of YY1-EZH2 interaction by the two peptides would be unlikely to alter their stability. Overall, we predict that the disruption of YY1-EZH2 interaction by either YPB or OPB peptide would cause significant gene expression perturbation in breast cancer cells, leading to their apoptotic cell death.

EZH2 mutations have been frequently reported in certain cancer types, including leukemia, lymphoma, and melanoma [48,54,55]. Most of these mutations have not been associated with physiological effects of the malignancies, although those identified in the SET domain could alter the methyltransferase activity of EZH2. Interestingly, the R502 of EZH2, a residue in the YPB domain, was also mutated in several cases of acute myeloid leukemia to generate an R502Q mutant [54]; however, whether this mutation alters the YY1-EZH2 interaction needs to be elucidated in future studies.

PTENP1 is a tumor suppressor gene that displayed reduced H3K27me3 signal when treated by YPB and OPB peptides. Previous studies revealed the role of PTENP1 as a ceRNA to trap microRNAs and consequently relieve their inhibition to the mRNA of the PTEN, a well-characterized tumor suppressor [45]. Various studies demonstrated the activity of PTENP1 in inhibiting breast cancer cell proliferation through promoting PTEN expression [56,57,58]. Consistently, PTEN and PTENP1 downregulation was significantly associated with poor prognosis of breast cancer patients [56].

In our study, we detected the upregulation of both PTENP1 and PTEN in the YPB and OPB peptide treated cells, and observed reduced S473 and T308 phosphorylation of AKT, suggesting its attenuated activation. It is noteworthy that the reduced AKT activation could be influenced by both PTENP1/PTEN upregulation and the blocked YY1-AKT interaction. To evaluate the contribution of PTENP1 to reduced breast cancer cell viability in response to the YPB and OPB peptides, we generated two shPTENP1 constructs, which reduced PTENP1 to about 40% of its original level, and carried out the cotreatments by these shRNAs and peptides. The YPB and OPB peptide treatments could reduce the H3K27me3 in the region of the PTENP1 promoter and consequently activate its gene expression. Thus, the two peptides could increase PTENP1 transcription, which counteracted the knockdown mediated by PTENP1 shRNAs and partially restored PTENP1 expression.

As a multifunctional transcription factor, YY1 can recruit many cofactors, including p300/CBP, HDACs, PRMTs, DNMTs, etc. to either activate or repress gene expression [25]. Therefore, EZH2 is not the only YY1-recruited cofactor, nor does it participate in YY1-mediated expression of all genes. It was thus not surprising that many reported YY1 target genes, including SNAI1, TP73, EGFR, CCL8, etc. [25], did not show H3K27me3 alterations in our ChIP-seq studies (data not shown). We certainly cannot exclude that reduced EZH2 binding may affect YY1-mediated recruitment of other cofactors. Thus, we predict that disrupted YY1-EZH2 interaction, by either YPB or OPB peptide, would cause significant gene expression perturbation in breast cancer cells with PTENP1 as a primary target, leading to apoptotic cell death.

Overall, our study discovered the YPB domain on EZH2 responsible for YY1 binding. Based on our previous finding of YY1′s OPB domain responsible for its binding of EZH2 and several other oncoproteins, we demonstrated the activities of synthetic YPB and OPB peptides in inhibiting the proliferation of breast cancer cells, but not nontumorigenic cells, and reducing tumor growth in a xenograft mouse model. Our data indicated that disruption of YY1-EZH2 interaction represents a new strategy in cancer therapies.

## 5. Conclusions

In this study, we mapped the YY1 protein binding (YPB) domain on the EZH2 protein to a region consisting of 27 residues. Synthetic peptides based on the YPB and OPB domain sequences could induce apoptosis of breast cancer cells and inhibit breast tumor growth in a xenograft mouse model. Using ChIP-seq analysis, we demonstrated that YPB and OPB peptides could decrease H3K27 trimethylation of the PTENP1 gene, and the upregulated PTENP1 expression was a primary reason of reduced proliferation of breast cancer cells caused by the treatments of the two peptides. Therefore, our study strongly suggests that the YPB and OPB peptides are potential therapeutics in cancer treatments.

## Figures and Tables

**Table 1:** Summary of GO and KEGG pathway enrichment analyses of the H3K27me3 changes in MDA-MB-231 cells treated by YPB and OPB peptides versus Cont peptide.

GO Enrichment			
Peptide (Gene Numbers)	Biological Process (BP)	Cell Component (CC)	Molecular Function (MF)
**YPB** (465)	Sensory (eye and ear) system development, neuron migration.	Synaptic membrane and other membrane structures.	Voltage-gated ion channel activity.
**OPB** (1137)	Neural and reproduction system development.	Cell-cell junction, adhesion, neuronal cell development, microvillus.	Membrane channel activities, including ion and lipid transmembrane transportation.
KEGG Pathway Enrichment			
**YPB** (465)	cAMP, MAPK, adrenergic, calcium, oxytocin signaling and cGMP−PKG pathways; regulations related to cardiac muscle, cholinergic synapse, and protein digestion and absorption.		
**OPB** (1137)	RAP1, WNT, HIPPO and thyroid hormone signaling pathways; breast cancer, gastric cancer, liver cancer, and stem cell regulations.		

## Data Availability

H3K27me3 Chip-seq experiments of MDA-MB-231 cells treated by YPB and OPB peptides were conducted in this study, and the datasets are available here: https://www.ncbi.nlm.nih.gov/geo/query/acc.cgi?acc=GSE171954, 16 April 2021. EZH2 and YY1 Chip-seq data were constructed using publicly available ENCODE datasets [44]. Accession numbers for all ENCODE datasets can be found in Supplementary Figure S5. Other experimental data can be found in the manuscript and Supplementary Materials.

## Supplementary Materials

The following are available online at https://www.mdpi.com/article/10.3390/cancers13102402/s1, Figure S1: Evaluation of protein-protein interaction using the surface plasmon resonance (SPR) analysis, Figure S2: Cotreatments of MDA-MB-231 cells by YPB/OPB peptide and doxorubicin (DOX), Figure S3: Integrative analyses of currently available YY1 (A) and EZH2 (B) ChIP-seq datasets in the ENCODE database, Figure S4: Effects of YY1 (A) and EZH2 (B) knockdown on the PTENP1 and PTEN gene expression in MDA-MB-231 cells, Figure S5: Aligned gene browser tracks between the H3K27me3 ChIPseq dataset from the YPB/OPB-treated MDA-MB-231 cells and two EZH2 ChIP-seq datasets from other cell lines, Figure S6: The images of all original Western blots, Table S1: Genes with the same (increased or decreased) trends of H3K27me3 alterations in MDA-MB-231 cells treated by YPB and OPB peptides versus Cont peptide in the ChIP-seq studies.
