# Supplementary material for: Disruption of YY1-EZH2 Interaction Using Synthetic Peptides Inhibits Breast Cancer Development
Source: Cancers (Basel). 2021 May 16;13(10):2402. doi: 10.3390/cancers13102402 (PMC8156467; doi:10.3390/cancers13102402)
Supplement: Supplementary file 1 [file cancers-13-02402-s001.zip › Supplementary Figure 1.pdf]

Supplementary Figure 1.

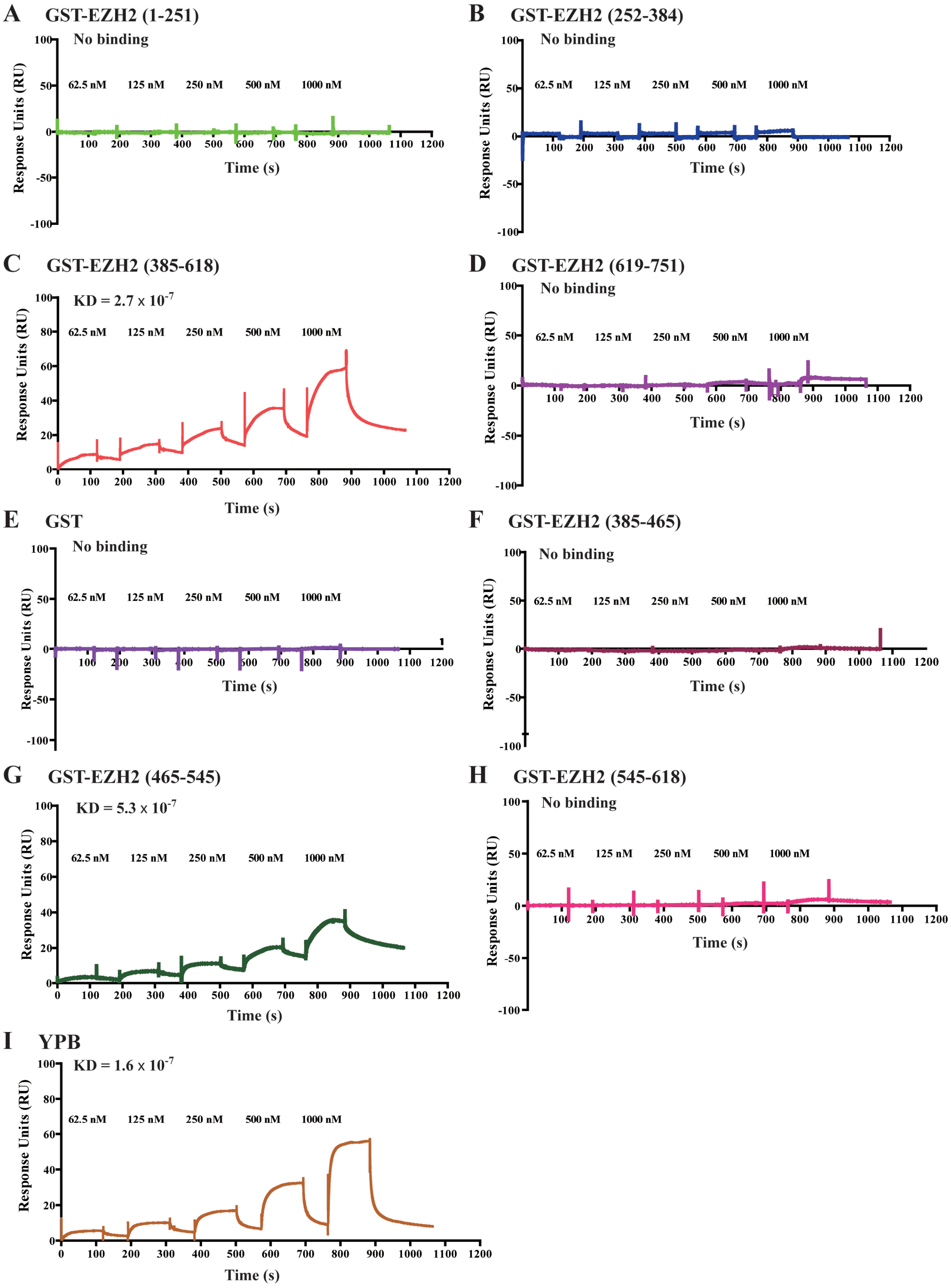

Supplementary Figure 1. **Evaluation of protein-protein interaction using the surface plasmon resonance (SPR) analysis.** The purified His×6-YY1 was conjugated to the

carboxymethylated dextran matrix of the CM5 chip (GE Healthcare). Purified GST-EZH2 mutant proteins (A, B, C, D, F, G and H), GST (E) and the YPB peptide (I) were serially diluted into serial concentrations of 62.5, 125, 250, 500 and 1000 nM for injection. The samples individually flowed over the chip surface, with the response units (RU) measured at a single cycle. The binding kinetics were analyzed using 1:1 binding model with Biacore T200 Evaluation software, version 2.0. The binding profiles are shown with time (s) as the x axis and the RU as the y axis.
