# Supplementary material for: Disruption of YY1-EZH2 Interaction Using Synthetic Peptides Inhibits Breast Cancer Development
Source: Cancers (Basel). 2021 May 16;13(10):2402. doi: 10.3390/cancers13102402 (PMC8156467; doi:10.3390/cancers13102402)
Supplement: Supplementary file 1 [file cancers-13-02402-s001.zip › Supplementary Figure 2.pdf]

Supplementary Figure 2.

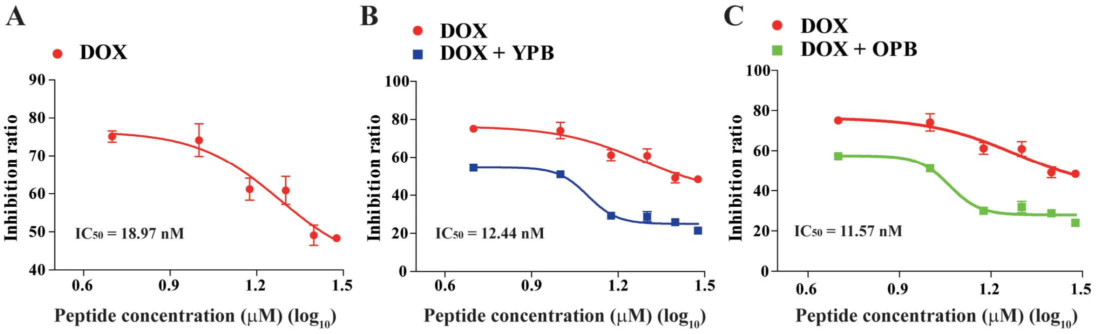

Supplementary Figure 2. Cotreatments of MDA-MB-231 cells by YPB/OPB peptide and doxorubicin (DOX). MDA-MB-231 cells were treated by different concentrations (5, 10, 15, 20, 25 and 30 nM) of DOX alone (A), in combination with 14.9 μM of YPB peptide (B), or 14.6 μM of OPB peptide for 48 h, followed by WST-1 assays to determine cell proliferation and calculation of inhibition ratios.
