# Supplementary material for: Disruption of YY1-EZH2 Interaction Using Synthetic Peptides Inhibits Breast Cancer Development
Source: Cancers (Basel). 2021 May 16;13(10):2402. doi: 10.3390/cancers13102402 (PMC8156467; doi:10.3390/cancers13102402)
Supplement: Supplementary file 1 [file cancers-13-02402-s001.zip › Supplementary Figure 3.pdf]

Supplementary Figure 3.

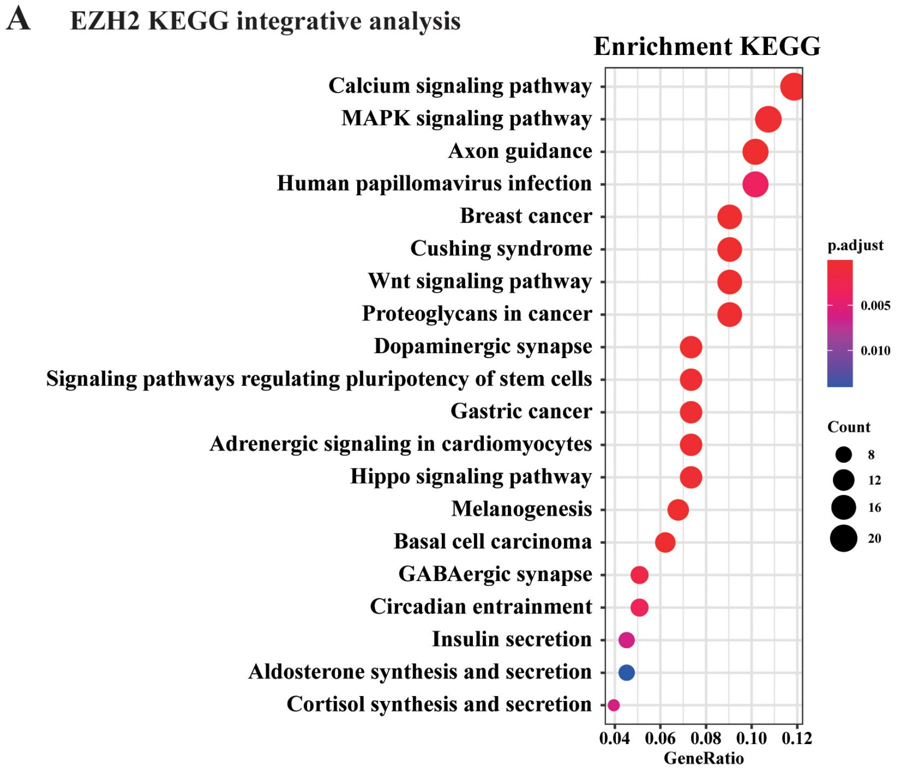

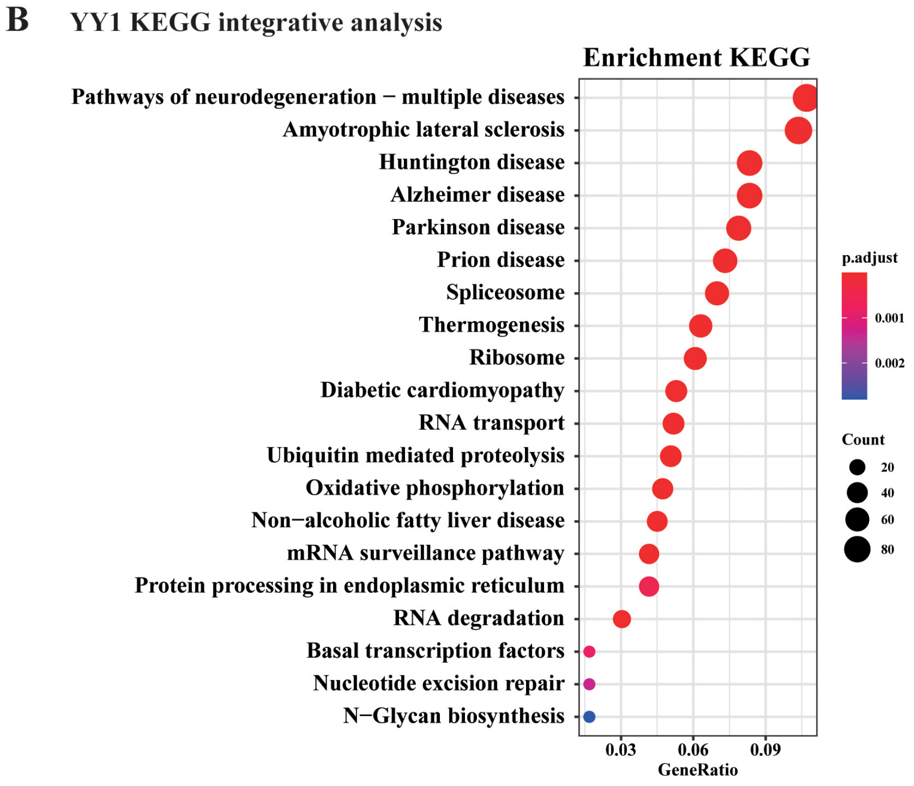

Supplementary Figure 3. Integrative analyses of currently available YY1 (A) and

EZH2 (B) ChIP-seq datasets in the ENCODE database. In A, integrative analyses of eight YY1 ChIP-seq datasets (ENCFF589PZO, ENCFF398UQZ, ENCFF352MLK, ENCFF042OEY, ENCFF958OUE, ENCFF246QIY, ENCFF567CJH and ENCFF545PFH) available in the ENCODE database (https://www.encodeproject.org/). In B, integrative analyses of two EZH2 ChIP-seq datasets (ENCFF521AKL and ENCFF545XSF) available in the ENCODE database. In both A and B, the top 20 GO terms of the KEGG pathway enrichment analyses of the integrative data are presented.
