# Supplementary material for: Disruption of YY1-EZH2 Interaction Using Synthetic Peptides Inhibits Breast Cancer Development
Source: Cancers (Basel). 2021 May 16;13(10):2402. doi: 10.3390/cancers13102402 (PMC8156467; doi:10.3390/cancers13102402)
Supplement: Supplementary file 1 [file cancers-13-02402-s001.zip › Supplementary Figure 4.pdf]

**Supplementary Figure 4.**

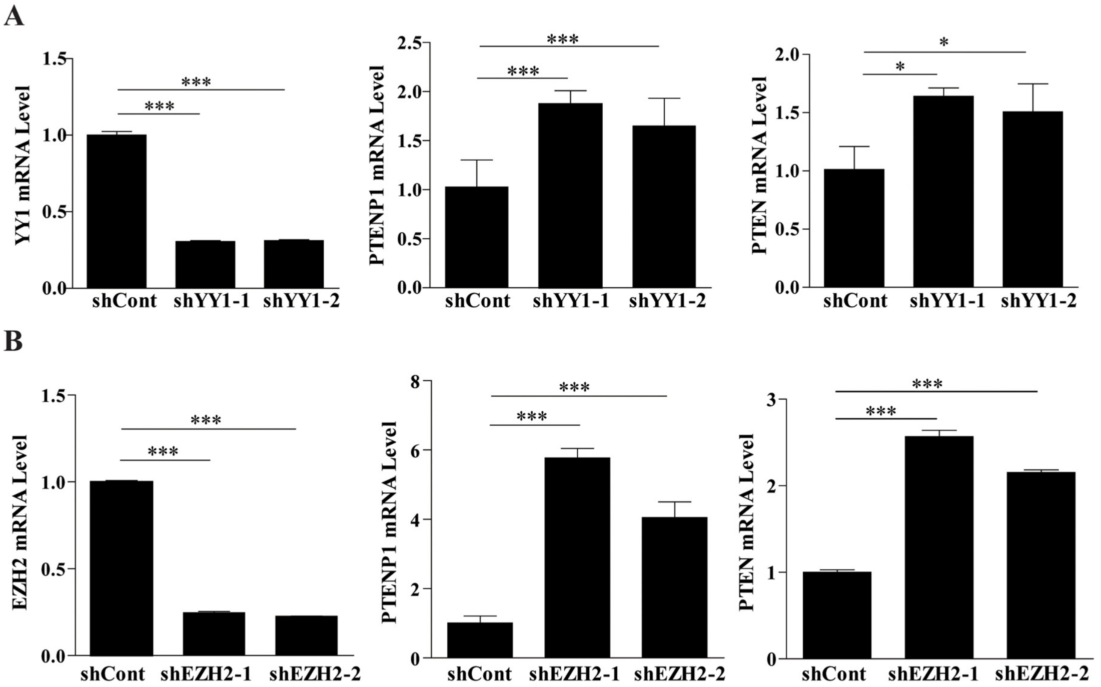


**Supplementary Figure 4. Effects of YY1 (A) and EZH2 (B) knockdown on the PTENP1 and PTEN gene expression in MDA-MB-231 cells.** MDA-MB-231 cells were infected by lentiviruses carrying shCont, shYY1-1 and shYY1-2 (in A), or shCont, shEZH2-1 and shEZH2-2 (in B). After 48 h, cells were cultured in medium containing 1 μg/ml of puromycin. After another 3 days of culture in the puromycin-containing medium, total RNAs were extracted from the cells, followed by qPCR to determine PTENP1 and PTEN expression, as well as YY1 (A) and EZH2 (B) mRNA levels.
