# Supplementary material for: Disruption of YY1-EZH2 Interaction Using Synthetic Peptides Inhibits Breast Cancer Development
Source: Cancers (Basel). 2021 May 16;13(10):2402. doi: 10.3390/cancers13102402 (PMC8156467; doi:10.3390/cancers13102402)
Supplement: Supplementary file 1 [file cancers-13-02402-s001.zip › Supplementary Figure 5.pdf]

Supplementary Figure 5.

A

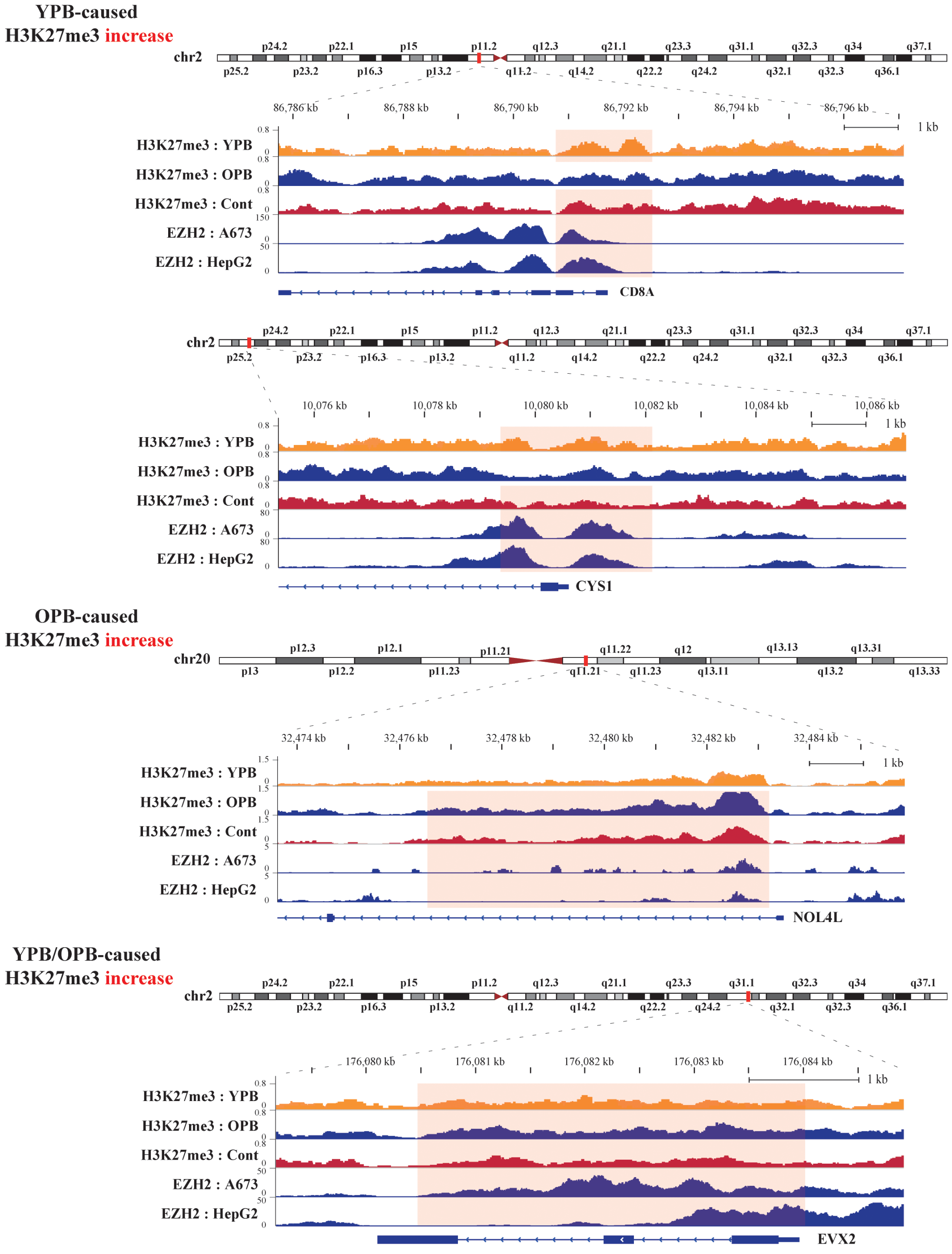

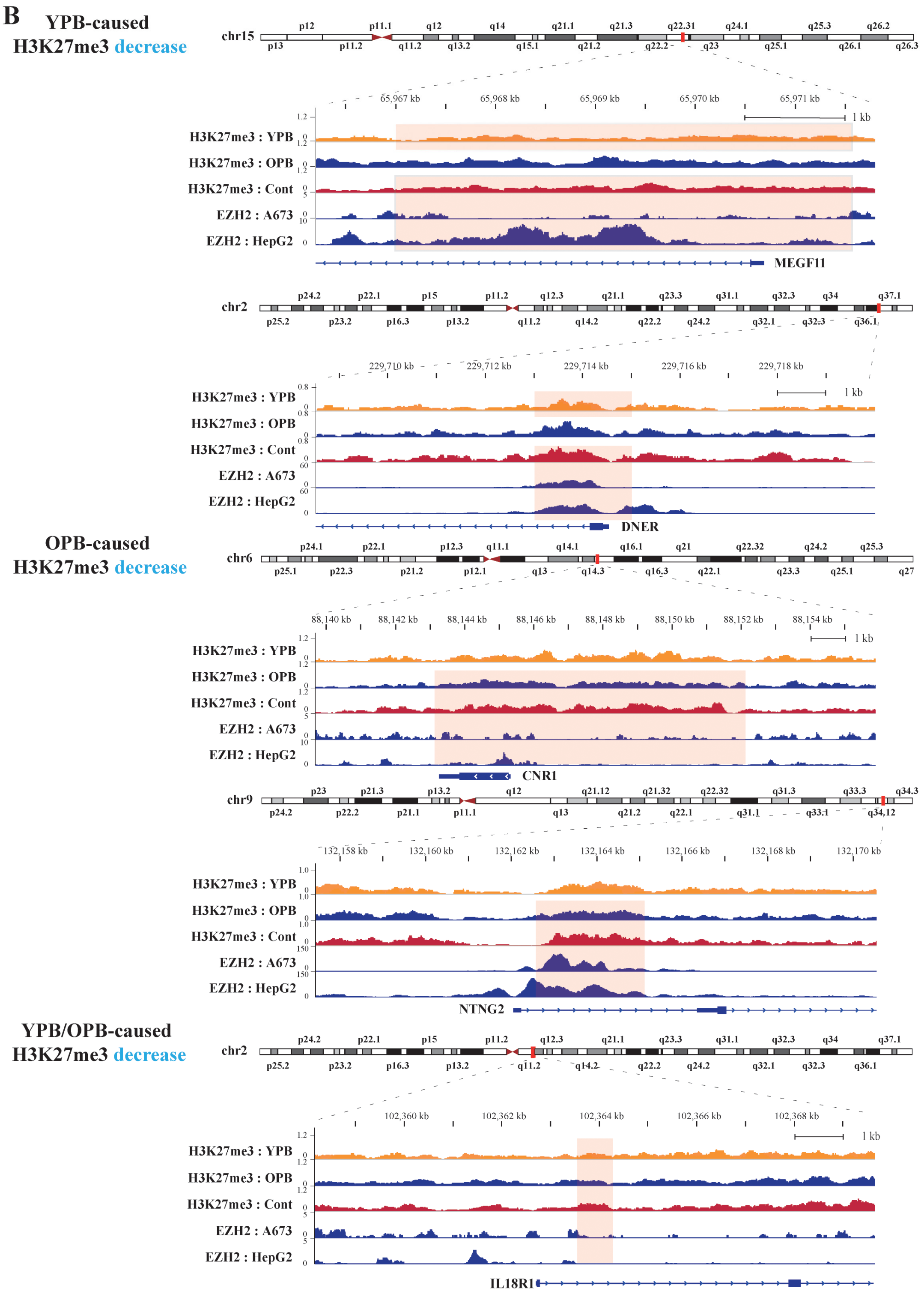
B
YPB-caused
H3K27me3 decrease
chr15
H3K27me3 : YPB
H3K27me3 : OPB
H3K27me3 : Cont
EZH2 : A673
EZH2 : HepG2
MEGF11
chr2
H3K27me3 : YPB
H3K27me3 : OPB
H3K27me3 : Cont
EZH2 : A673
EZH2 : HepG2
DNER
OPB-caused
H3K27me3 decrease
chr6
H3K27me3 : YPB
H3K27me3 : OPB
H3K27me3 : Cont
EZH2 : A673
EZH2 : HepG2
CNR1
chr9
H3K27me3 : YPB
H3K27me3 : OPB
H3K27me3 : Cont
EZH2 : A673
EZH2 : HepG2
NTNG2
YPB/OPB-caused
H3K27me3 decrease
chr2
H3K27me3 : YPB
H3K27me3 : OPB
H3K27me3 : Cont
EZH2 : A673
EZH2 : HepG2
IL18R1

C

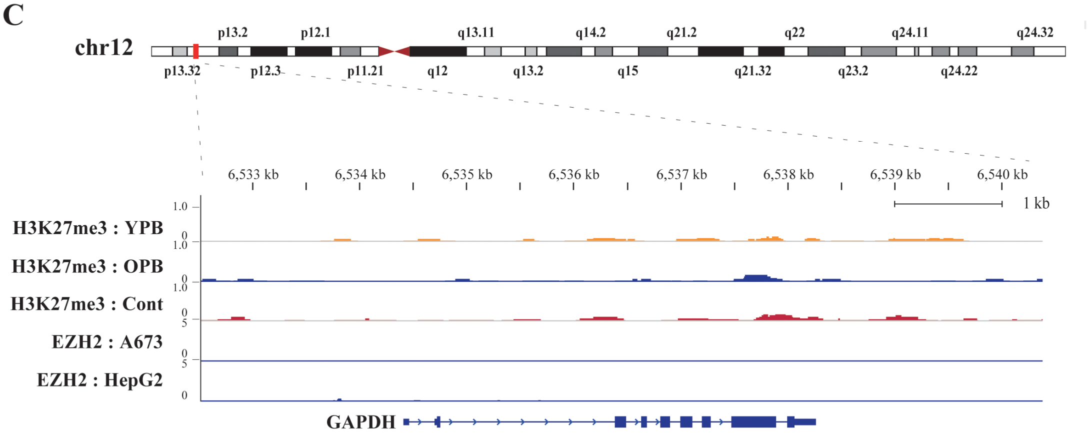

Supplementary Figure 5. Aligned gene browser tracks between the H3K27me3 ChIP-seq dataset from the YPB/OPB-treated MDA-MB-231 cells and two EZH2 ChIP-seq datasets from other cell lines. Both increased (A) and decreased (B) H3K27me3 signals caused by YPB, OPB or both treatments are presented, together with the EZH2 ChIP-seq signals from the A673 and HepG2 cells (ENCFF521AKL and ENCFF545XSF in the ENCODE database) at the same loci. The regions of the tracks with increased (in A) and decreased (in B) changes are marked with shadows. In C, the gene browser tracks of the GAPDH gene locus are presented.
