# Supplementary material for: Disruption of YY1-EZH2 Interaction Using Synthetic Peptides Inhibits Breast Cancer Development
Source: Cancers (Basel). 2021 May 16;13(10):2402. doi: 10.3390/cancers13102402 (PMC8156467; doi:10.3390/cancers13102402)
Supplement: Supplementary file 1 [file cancers-13-02402-s001.zip › Supplementary Figure 6.pdf]

Fig 1B

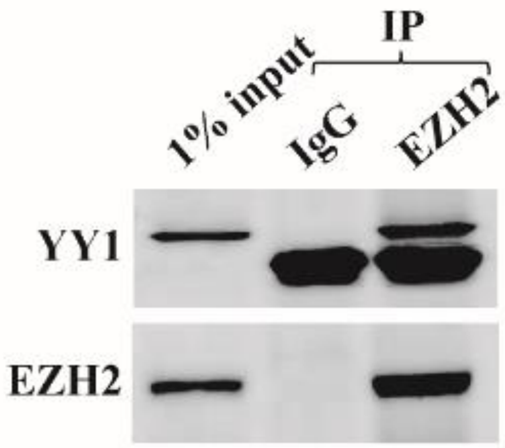

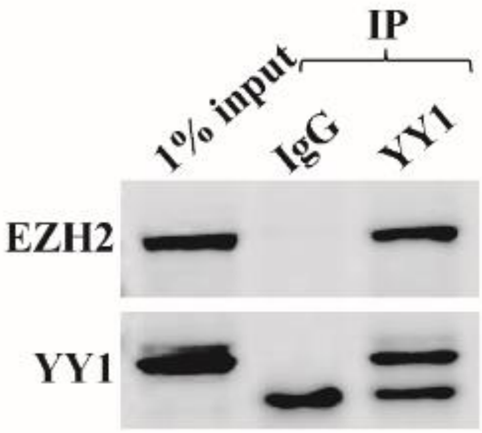

Original Western blot images

Original Western blot images

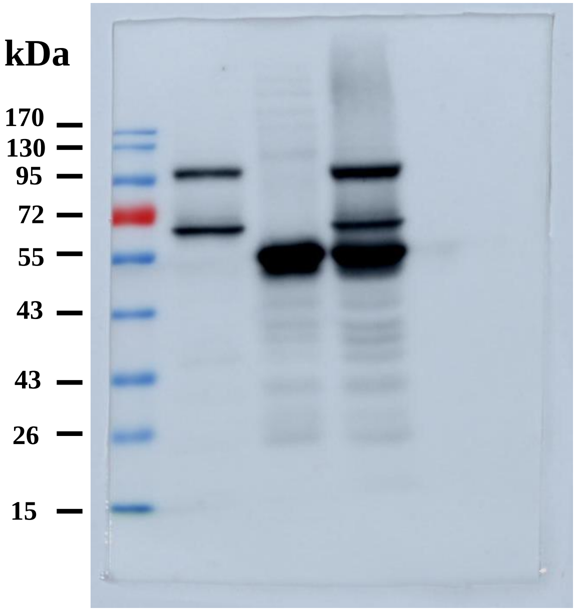

IP-EZH2- anti-YY1

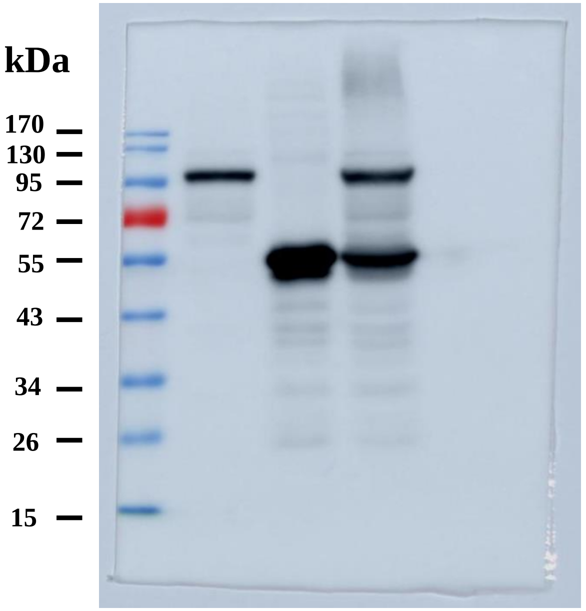

IP-EZH2- anti-EZH2

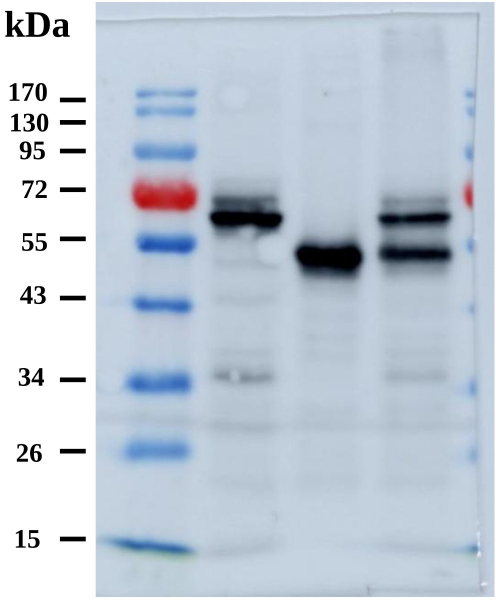

IP-YY1 anti-YY1

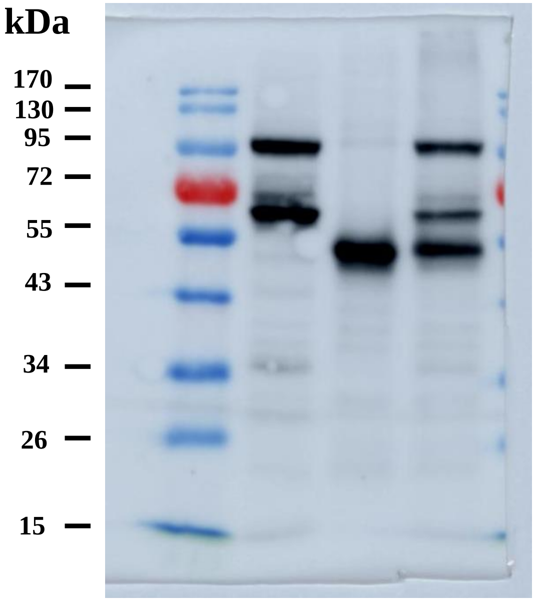

IP-YY1 anti-EZH2

Fig 1D

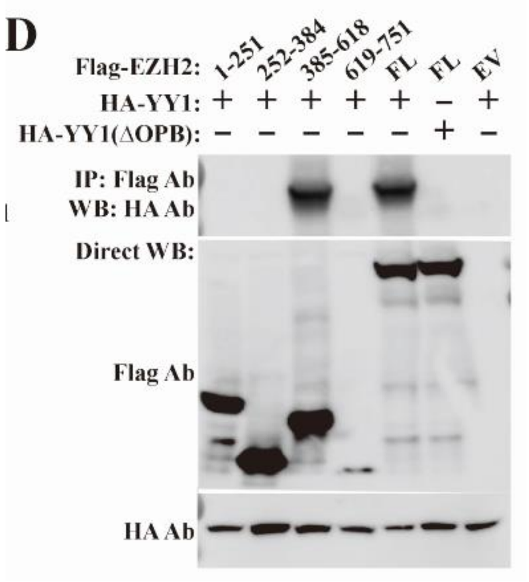

Original Western blot images

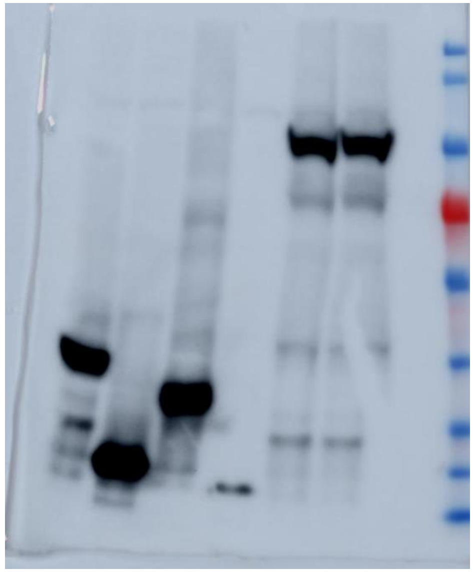

Input antibody Flag

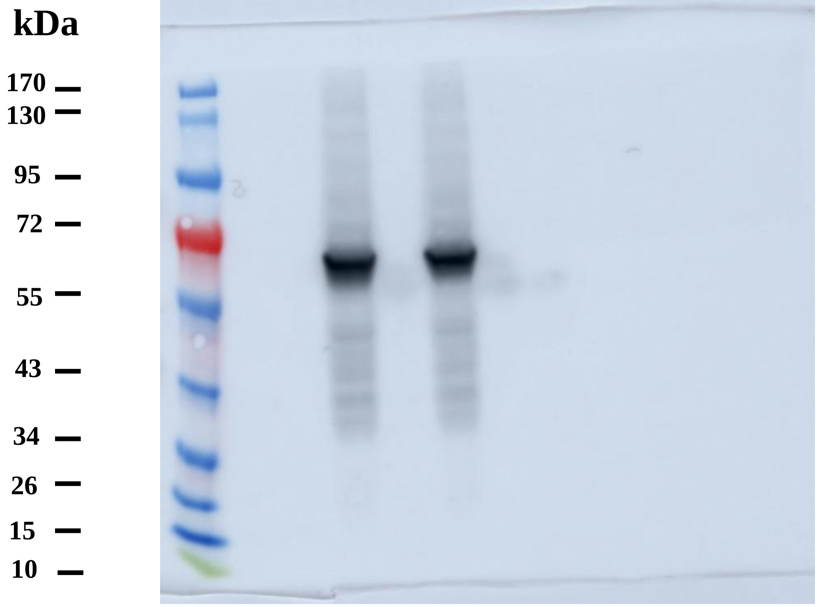

Antibody-HA

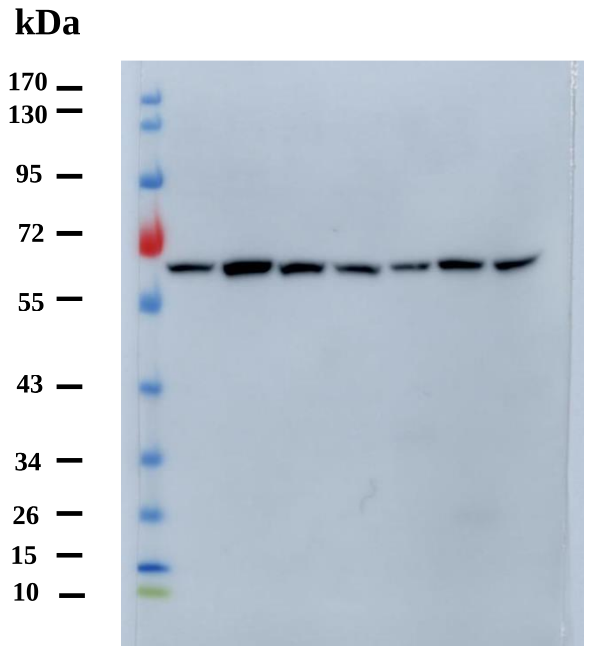

Input antibody HA

Fig 1E

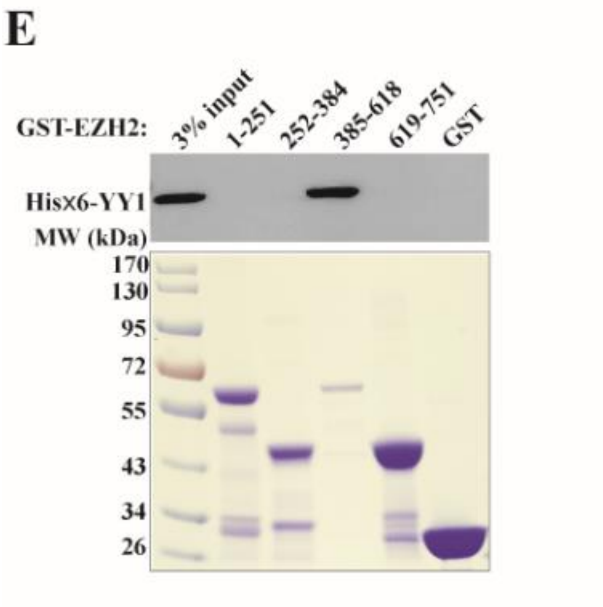

Original Western blot images

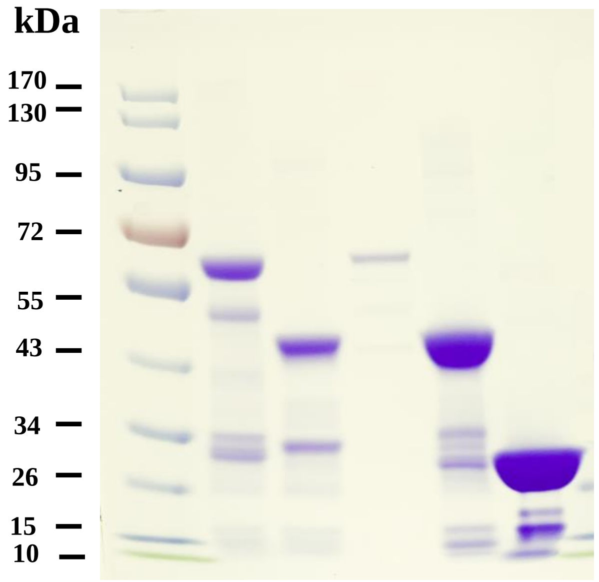

Coomassie brilliant blue staining

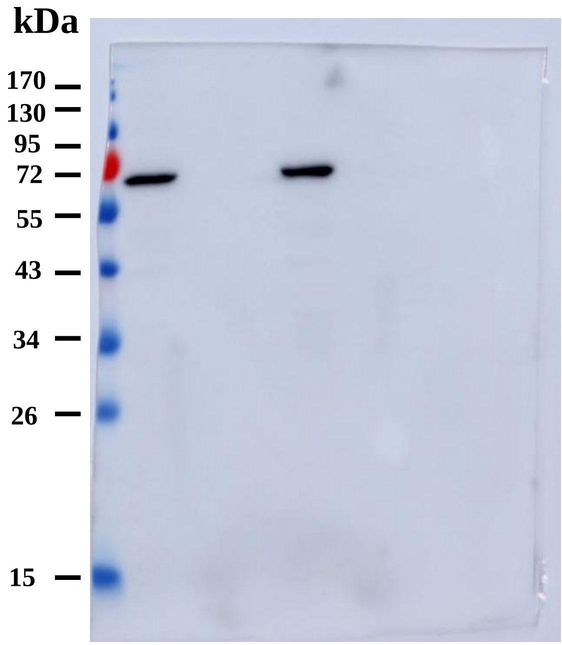

Antibody-His

Fig 1G

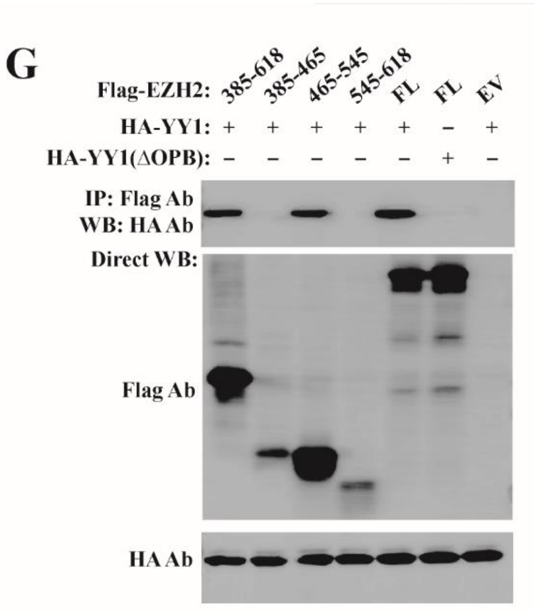

Original Western blot images

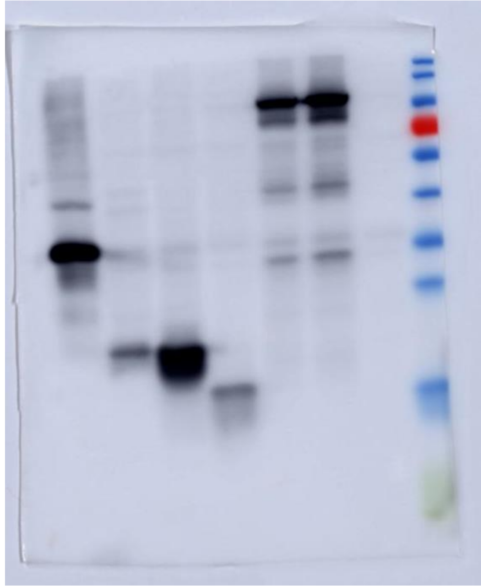

Input antibody Flag

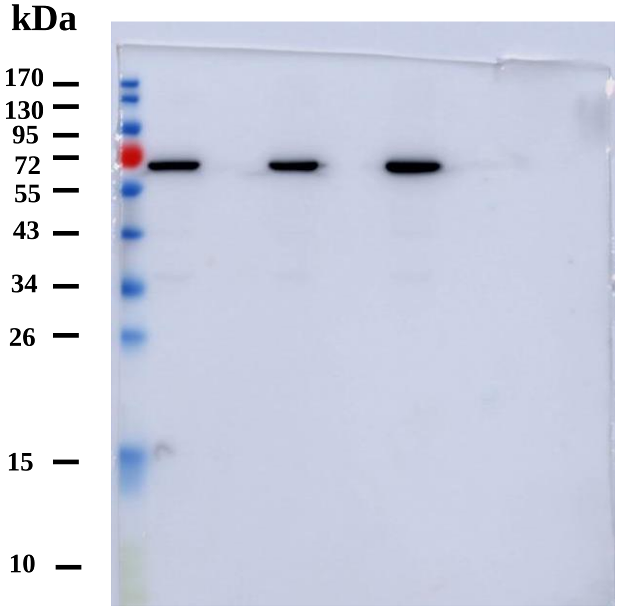

Antibody HA

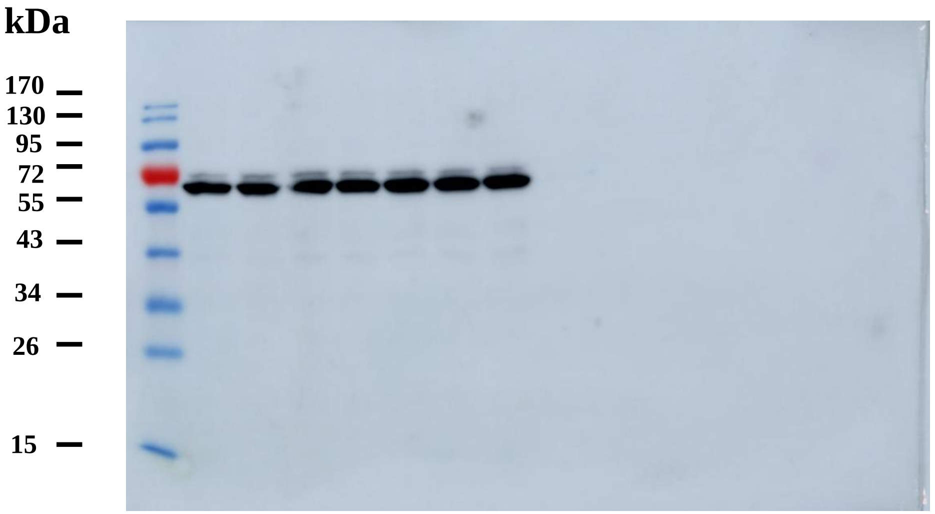

Input antibody HA

Fig 1H

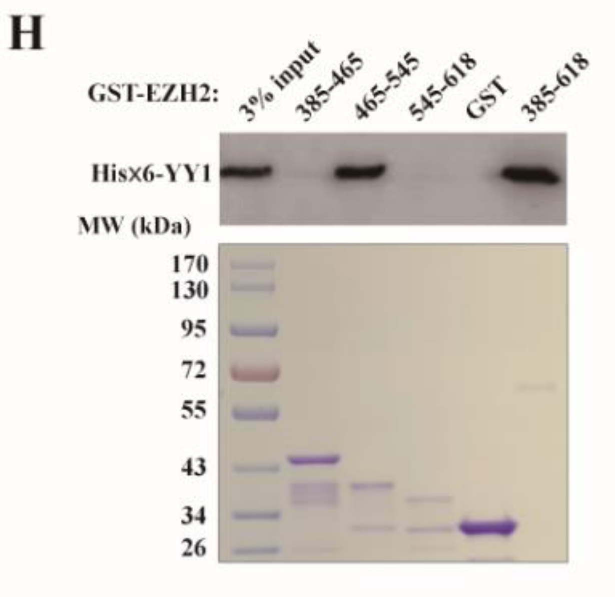

Original Western blot images

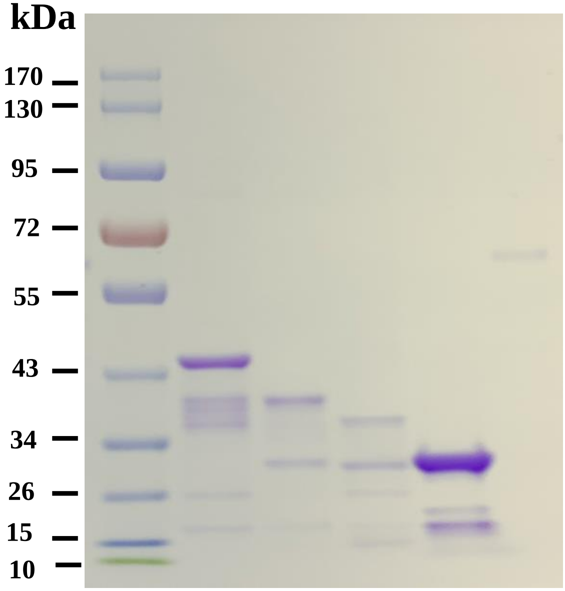

Coomassie brilliant blue staining

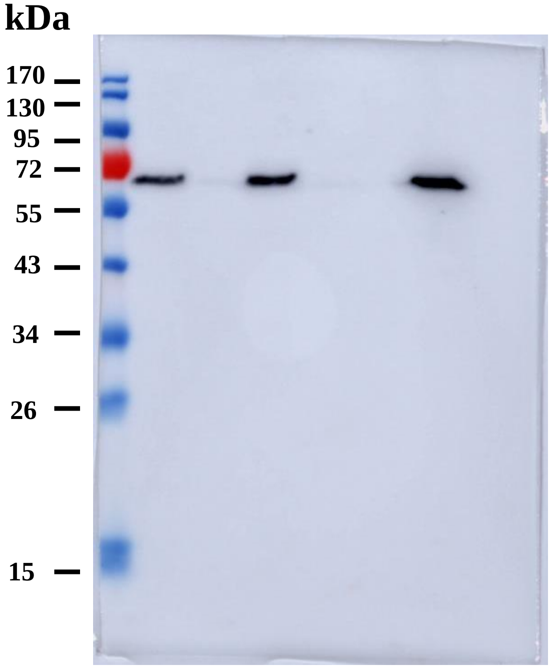

Antibody-His

Fig 1J

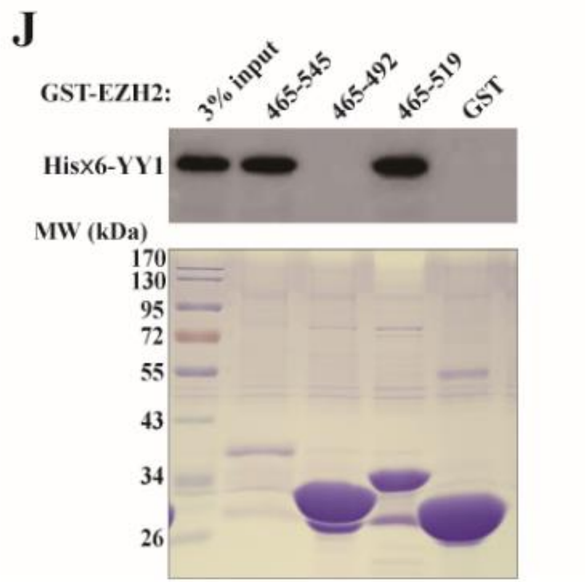

Original Western blot images

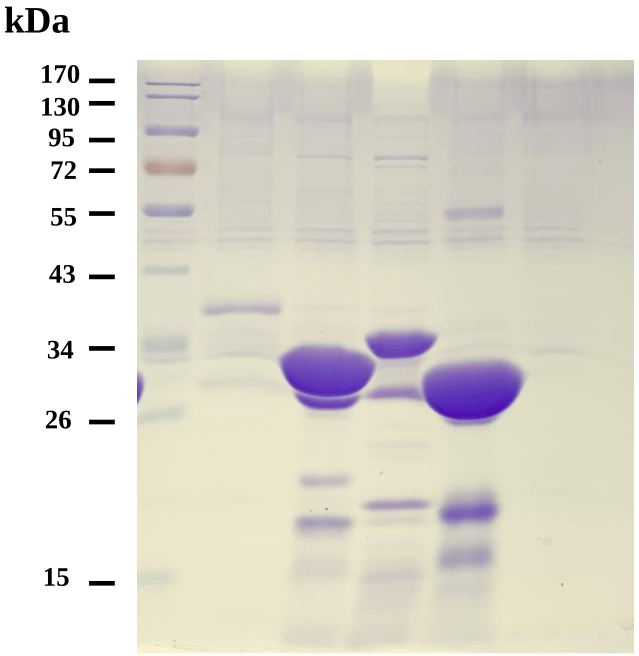

Coomassie brilliant blue staining

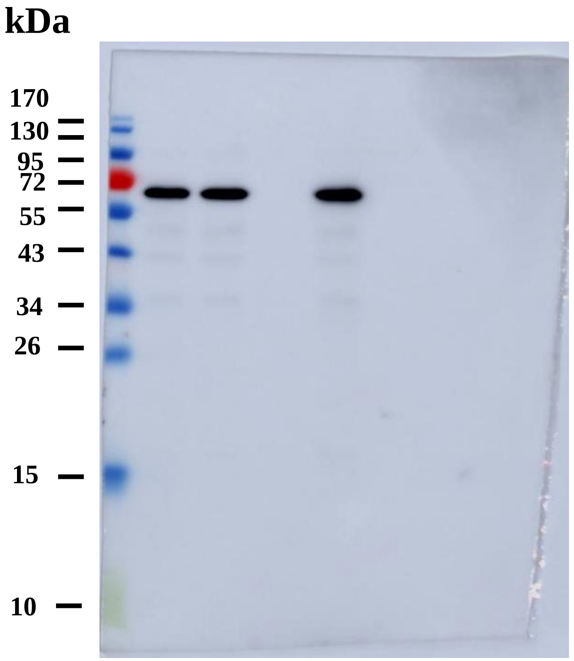

Antibody-His

Fig 4A

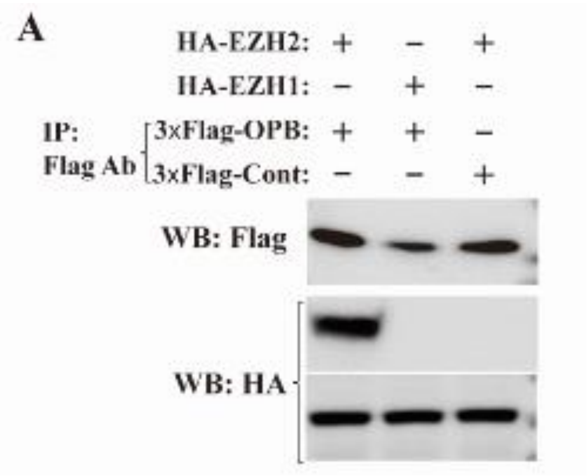

Original Western blot images

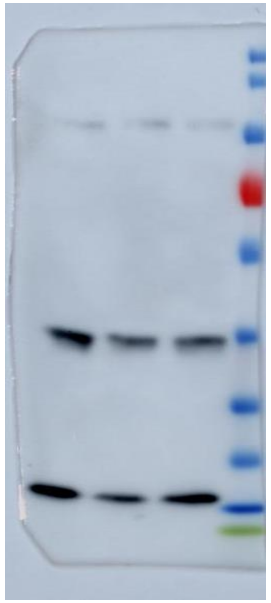

Input antibody Flag

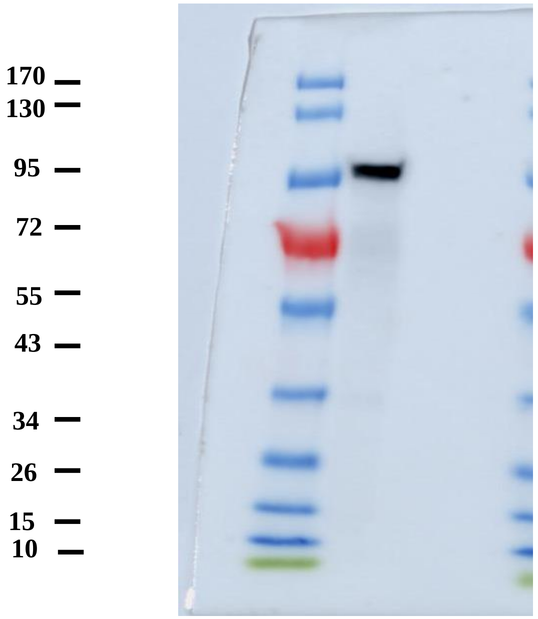

Antibody HA

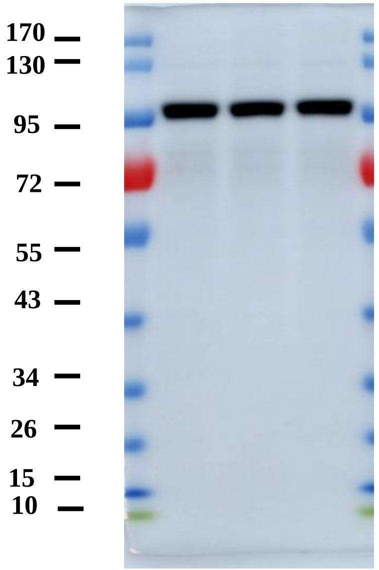

Input antibody HA

Fig 4B

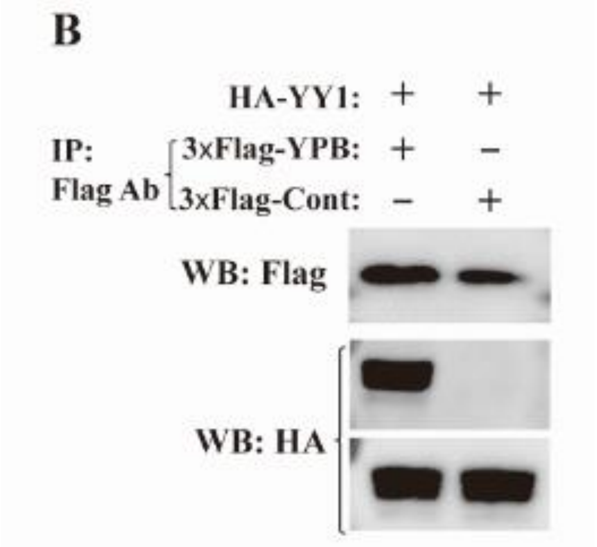

Original Western blot images

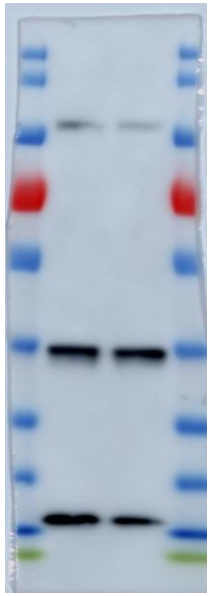

Input antibody Flag

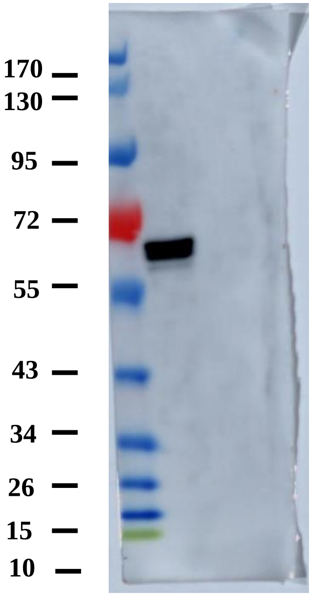

Antibody HA

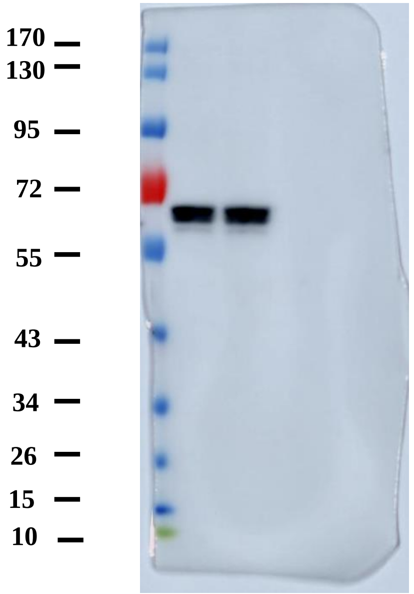

Input antibody HA

Fig 4C

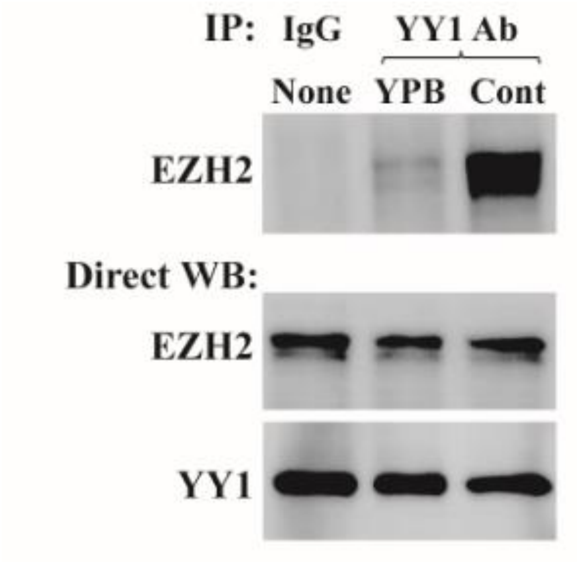

Fig 4D

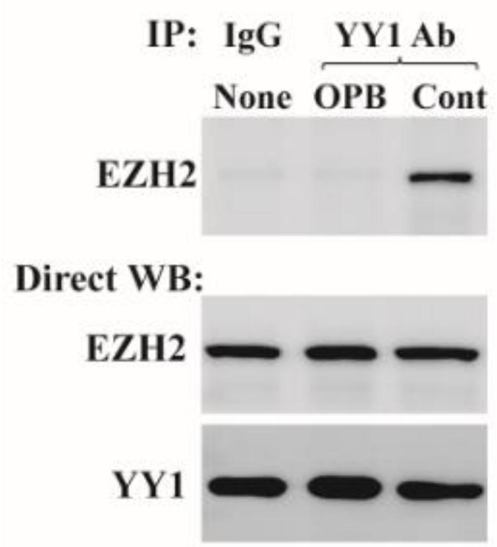

Original Western blot images

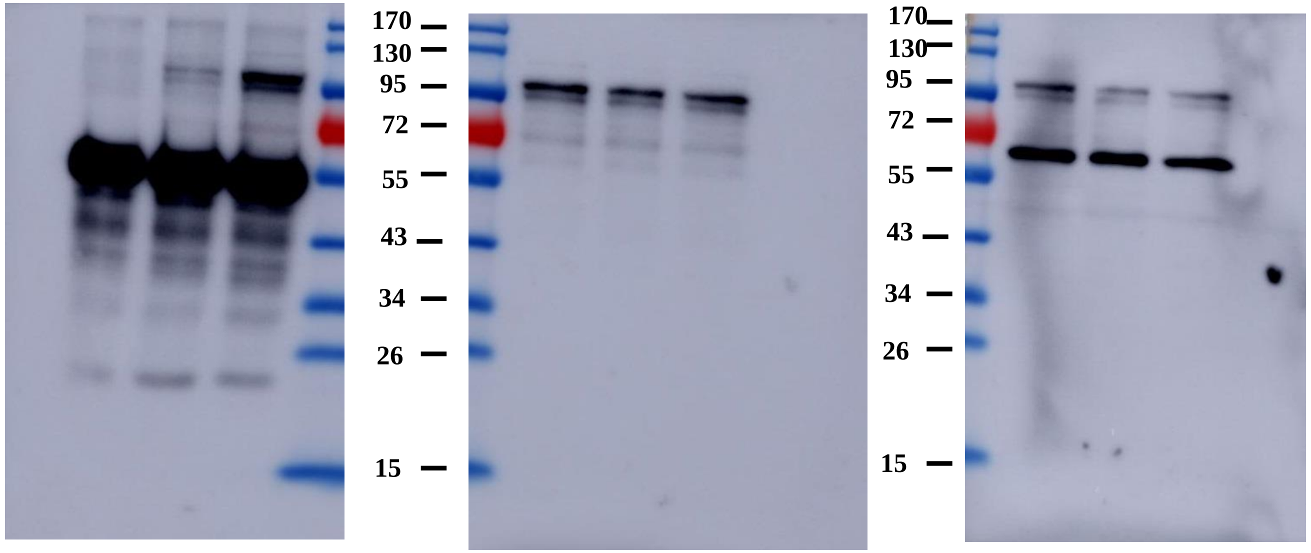

YPB IP-YY1-antibody-EZH2

YPB-Input-antibody-EZH2

YPB-Input-antibody-YY1

Original Western blot images

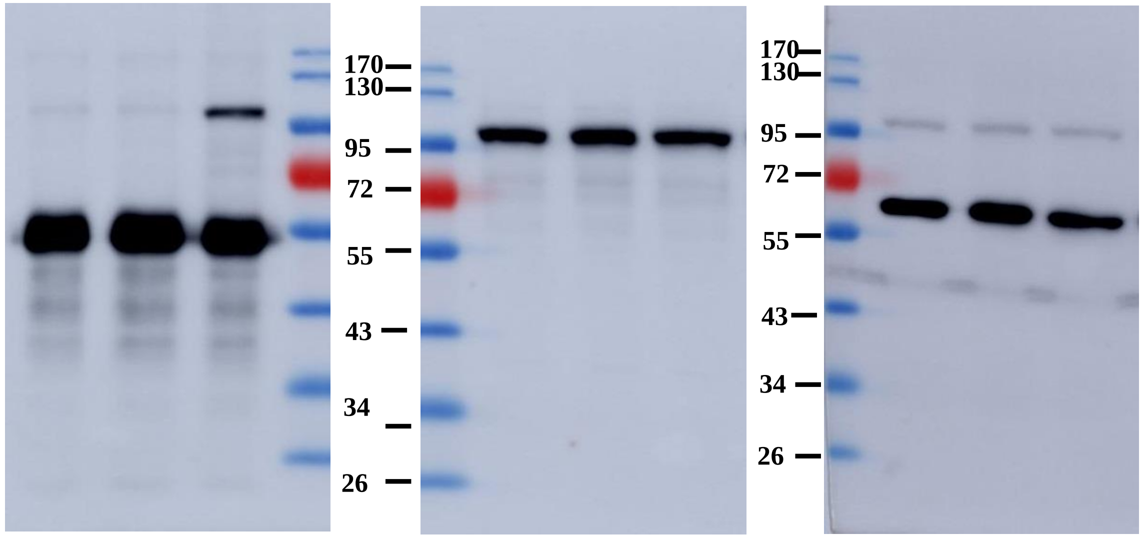

OPB-IP-YY1-antibody-EZH2

OPB-Input-antibody-EZH2

OPB-input -antibody-YY1

Fig 3E

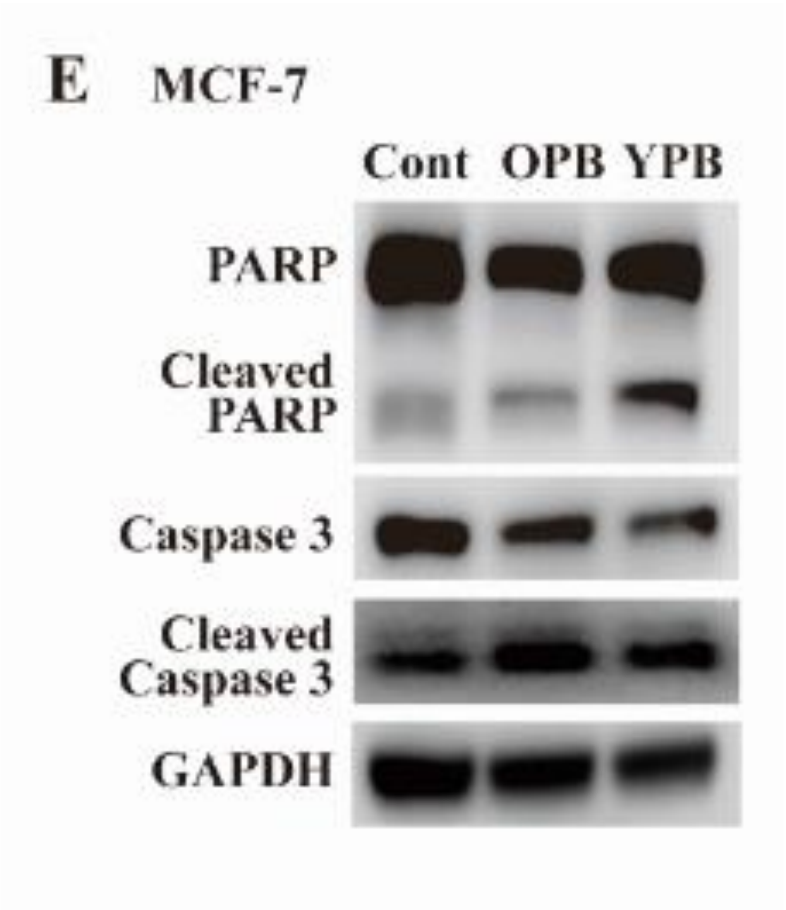

Original Western blot images

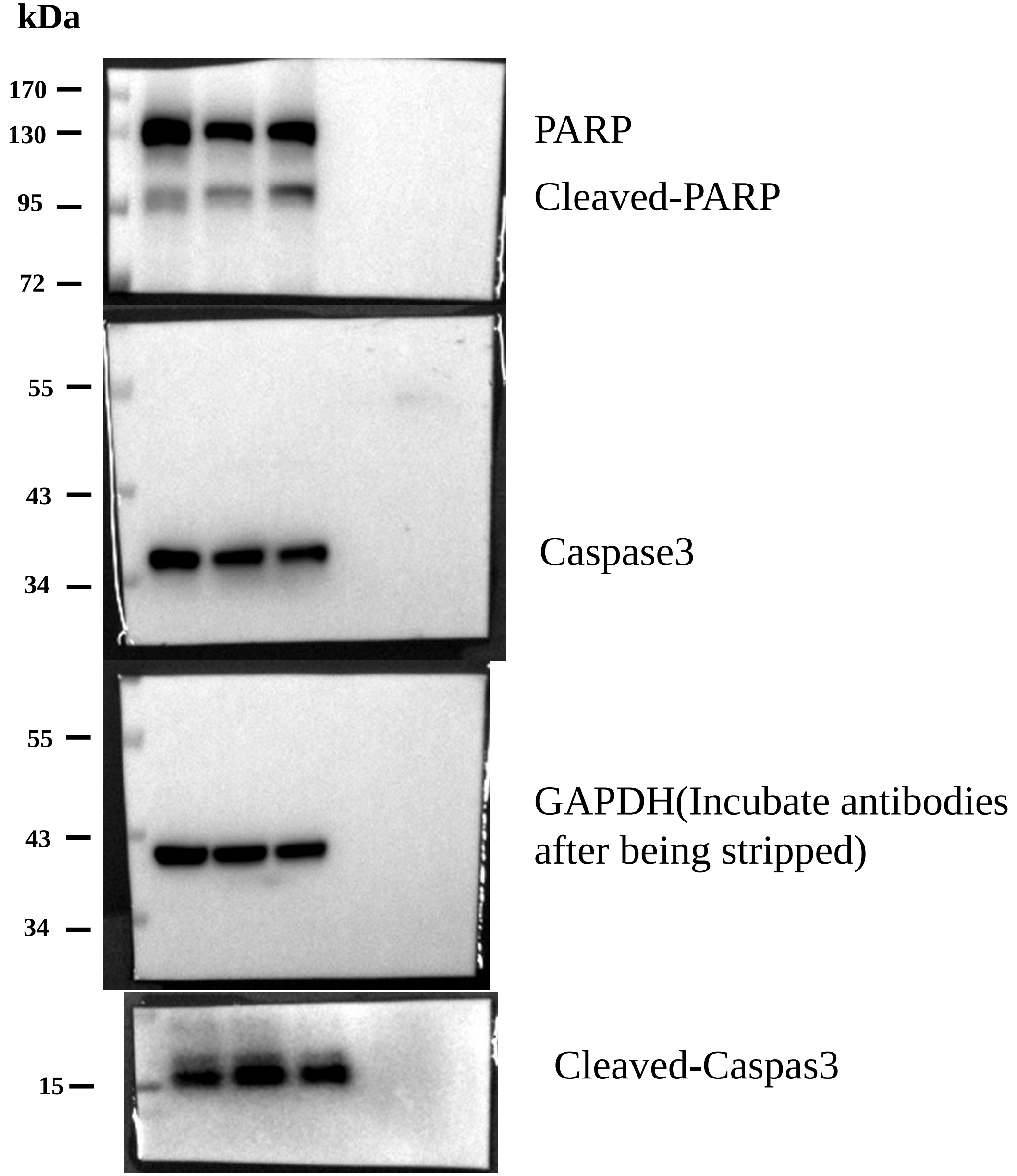

Fig 3F

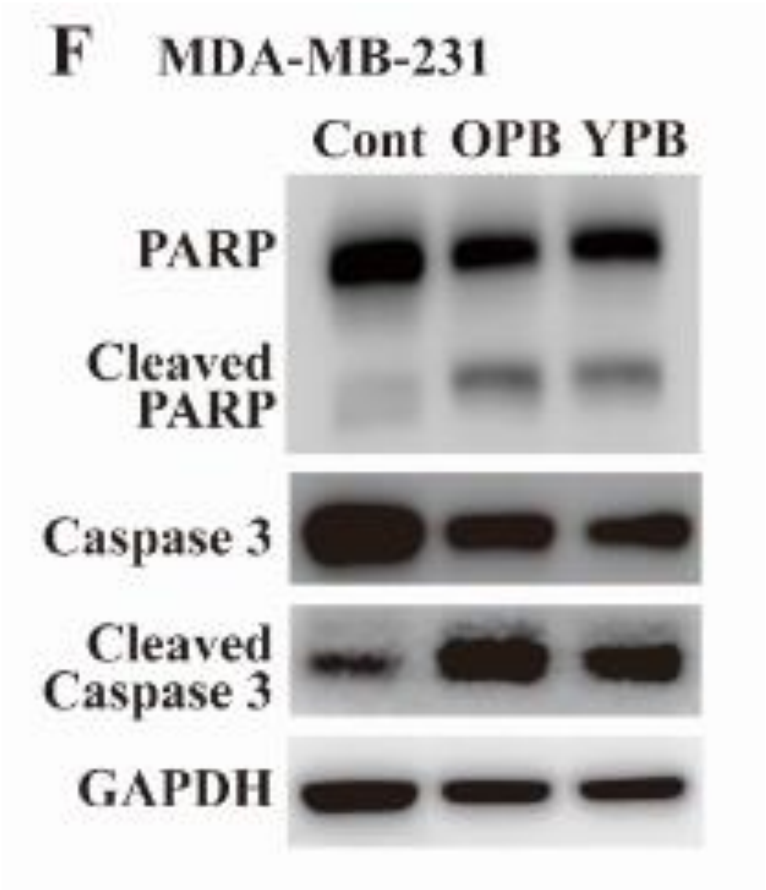

Original Western blot images

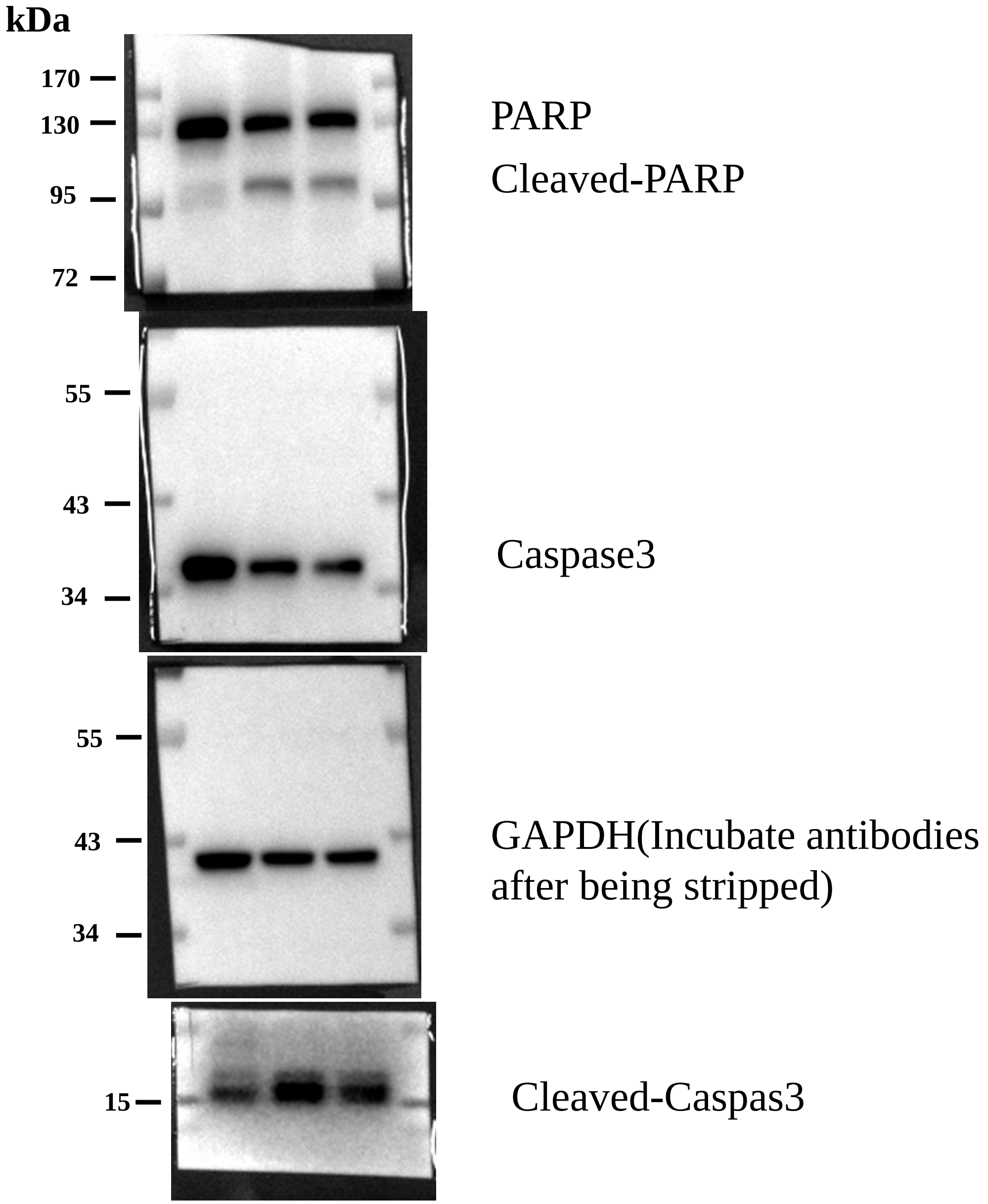

Fig 3G

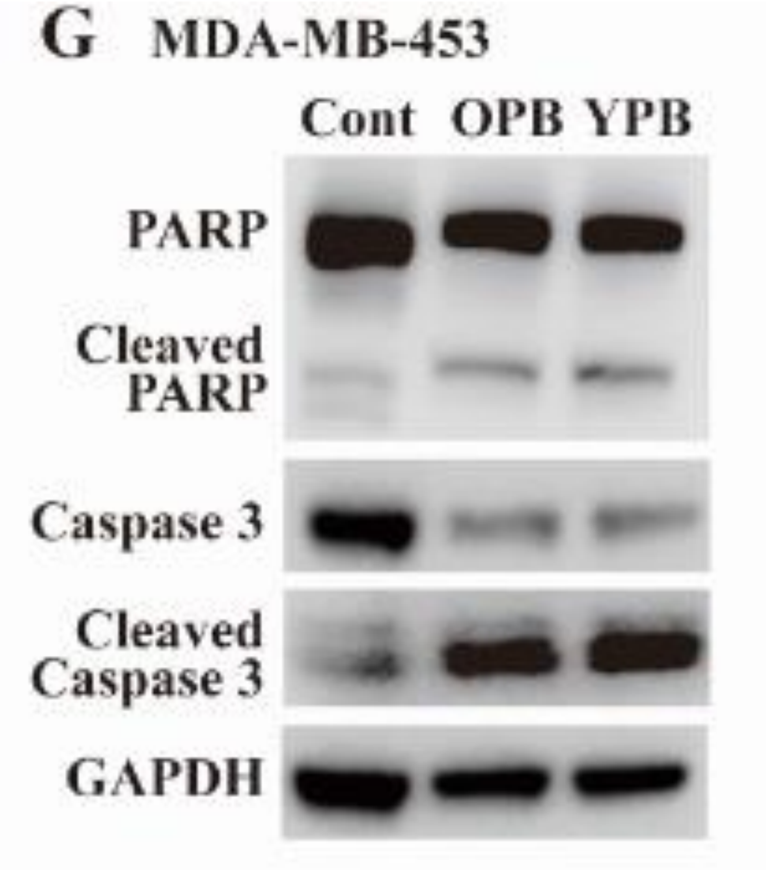

Original Western blot images

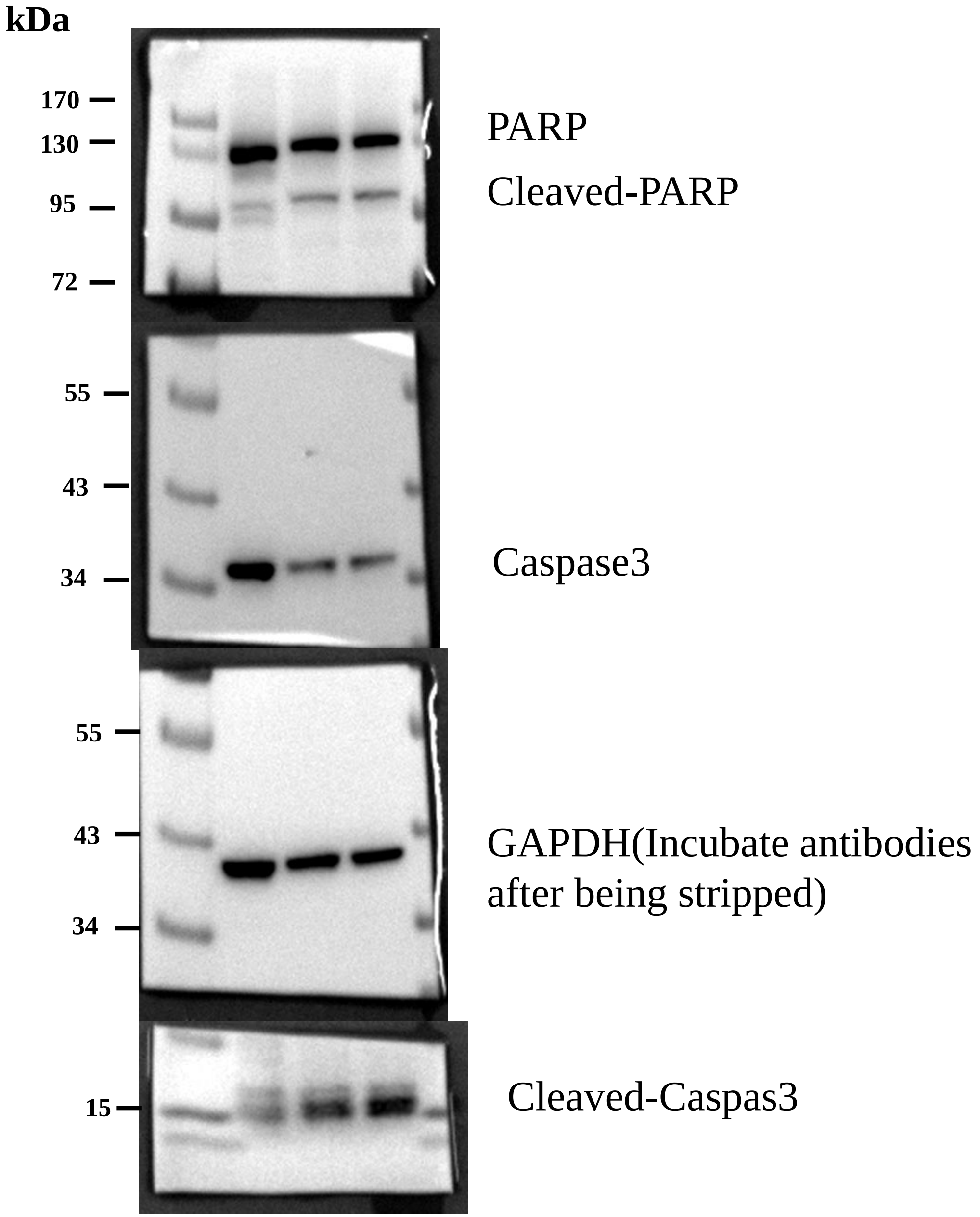

Fig 7C

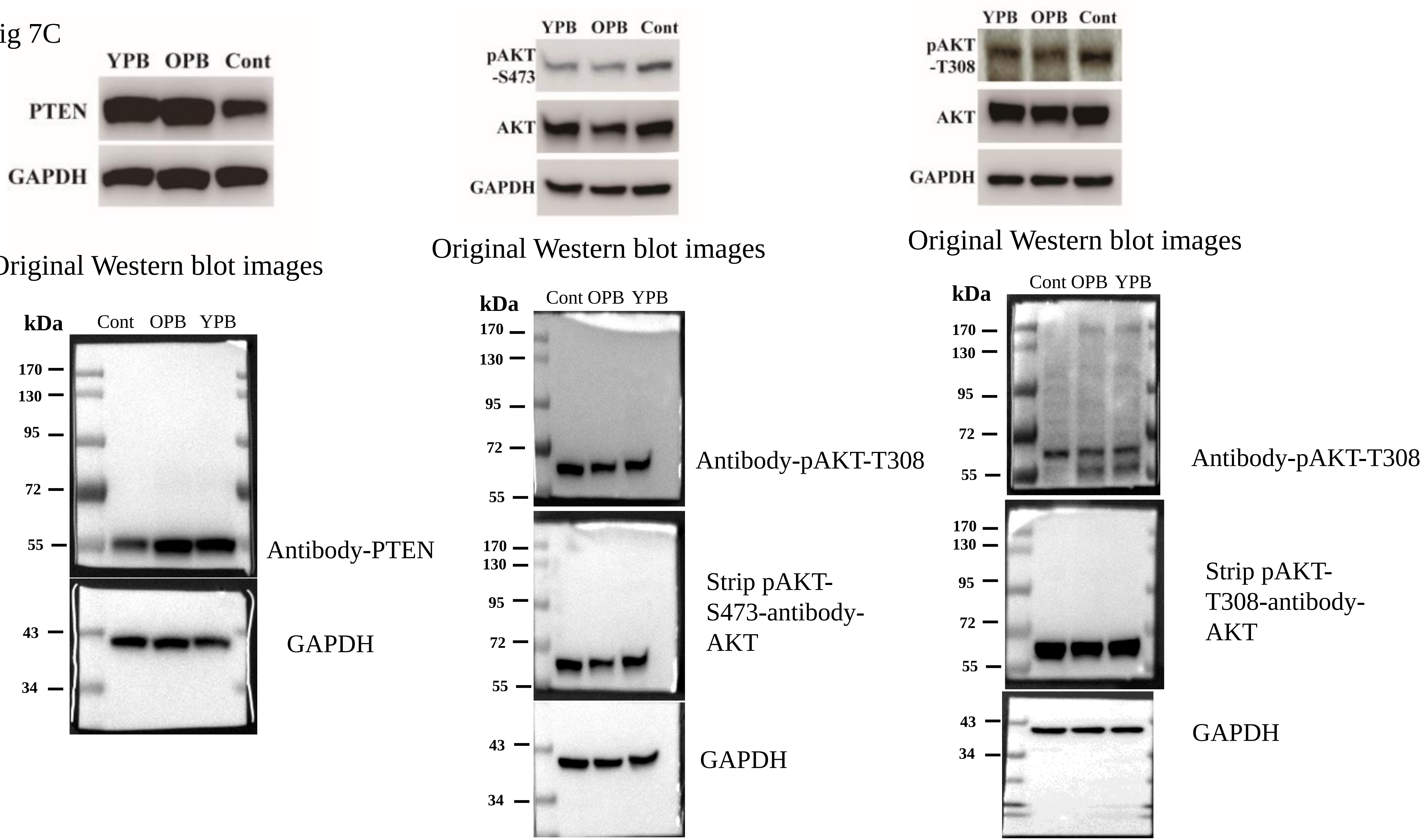
YPB OPB Cont
PTEN
GAPDH
YPB OPB Cont
pAKT -S473
AKT
GAPDH
YPB OPB Cont
pAKT -T308
AKT
GAPDH
Original Western blot images
kDa Cont OPB YPB
170
130
95
72
55
Antibody-PTEN
43
34
GAPDH
Original Western blot images
kDa Cont OPB YPB
170
130
95
72
55
Antibody-pAKT-T308
170
130
95
72
55
Strip pAKT-S473-antibody-AKT
43
34
GAPDH
Original Western blot images
kDa Cont OPB YPB
170
130
95
72
55
Antibody-pAKT-T308
170
130
95
72
55
Strip pAKT-T308-antibody-AKT
43
34
GAPDH
