# Supplementary material for: Disruption of YY1-EZH2 Interaction Using Synthetic Peptides Inhibits Breast Cancer Development
Source: Cancers (Basel). 2021 May 16;13(10):2402. doi: 10.3390/cancers13102402 (PMC8156467; doi:10.3390/cancers13102402)
Supplement: Supplementary file 1 [file cancers-13-02402-s001.zip › Supplementary Table 1.pdf]

Table 1. Genes with the same (increased or decreased) trends of H3K27me3 alterations in MDA-MB-231 cells treated by YPB and OPB peptides versus Cont peptide in the ChIP-seq studies.

	H3K27me3 ChIP signal	
	**Increased (93 genes)**	**Decreased** (38 genes)
**Gene names**	RAB11FIP4, SPNS3, PEAR1, HSD17B3, MYCBPAP, TSSK1B, CACNB3, TTLL6, SNED1, AC126915.1, RPSAP69, AC118754.1, C10orf105, EVX2, AC021491.1, RBP4, MATN2, DHRS3, GRK1, OR4R1P, EEF1A1P8, MEGF6, AC113348.1, DMKN, AC090955.1, YPEL2, MARK1, LINC00051, AC080078.1, CARNS1, FER1L4, AL049833.3, MUC21, SMAD6, RN7SL33P, PCSK9, OR4A42P, HCN2, KCNMA1, AC017033.1, DIPK1B, MIR551A, RASGEF1C, HCAR1, OSBPL5, RAPGEF3, AC093392.1, RHCG, AL390726.6, INPP5J, CASC15, PLEKHA7, CLPSL2, RNF183, TFEB, AC004080.1, AL354984.3, ATOH8, TMPRSS6, FBN3, AC087645.3, AC011840.1, NCS1, GSN, ENPP7, DEGS2, CLEC2L, PRKCZ, MFAP3, TMEM105, VASH1, GGT7, RAB15, RN7SKP280, GALNT16, AC091932.4, PMEPA1, PPY2P, PIK3C2B, KLHDC8A, LINC01978, AL096828.1, JAG2, GRHL3, AC025437.6, AC090825.1, PPP1R32, AL359853.1, LRRN4, AC244517.5, PPIE, UCMA, RARRES2P11.	PTENP1, RN7SKP73, FLT4, AC007638.1, SLCO2B1, LIFR, SCD, OR5B3, MYH10, TRIM26BP, RNU6-129P, AL162726.3, RNA5SP154, SLFN14, OXCT1, CLVS1, IFT81, CFAP221, CA11, AC002375.1, OR5W2, RNU6-662P, AC018697.1, LINC01866, KHDRBS3, RNU6-114P, AC134508.1, AC046168.2, HSPE1P1, IL18R1, ARMC3, KRT26, MIR3142HG, FAM153A, LAMA5, MAP7D2, HOOK1.
